# Mortality, Renal Replacement Therapy and Renal Impairment Markers in Critically Ill Patients in a Multinational Point-Prevalence ICU Cohort over 2007 to 2024: A nutritionDay Descriptive Multivariable Analysis

**DOI:** 10.3390/jcm15187119

**Published:** 2026-09-14

**Authors:** Arabella Fischer-Hammerschmied, Peter Bauer, Fiona Trumshi, Silvia Tarantino, Emir Softic, Christian Schuh, Sylvia Ryz, Martin H. Bernardi, Andrea Lassnigg, Johanna Tichy, Sarah Yadavalli, Anna Schranz, Michael Hiesmayr

**Affiliations:** 1 Division of Cardiothoracic and Vascular Anaesthesia and Intensive Care Medicine, Department of Anaesthesia, Intensive Care and Pain Medicine, Medical University of Vienna, 1090 Vienna, Austria; 2 Center for Medical Data Science, Institute of Medical Statistics, Medical University of Vienna, 1090 Vienna, Austria; 3 Ludwig Boltzmann Institute for Arthritis and Rehabilitation, Medical University of Vienna, 1090 Vienna, Austria; 4 IT Systems and Communications, Medical University of Vienna, 1090 Vienna, Austria

**Keywords:** renal replacement therapy, renal impairment, creatinine, urine volume, mortality, intensive care, critically ill, nutritionDay

## Abstract

**Background:** The study goal was to assess the association between mortality and renal impairment markers in the nutritionDay ICU cohort, incorporating creatinine, urine volume, and renal replacement therapy (RRT) while adjusting for ICU- and patient-level factors. **Methods:** During nutritionDay, participating ICUs worldwide prospectively documented all patients treated on a predefined study day in November. Renal impairment markers included creatinine and urine volume at ICU admission and RRT status on nutritionDay. ICU-level factors included world region, calendar year and time of assessment. Patient-level factors included sex, age, BMI, disease severity, and underlying disease leading to ICU admission. Sixty-day hospital mortality and use of RRT on nutritionDay were assessed in multivariable logistic regressions including all ICU- and patient-level factors. **Results:** The post hoc analysis included 23,485 adult ICU patients from 1855 ICU-year units in 60 countries from 2007 to 2024, with analytic sample sizes of 23,485 for RRT, 18,245 for mortality, 21,054 for creatinine, and 20,753 for urine volume. Mean age was 61.4 (SD 17.0) years. Mean SAPS 2 at ICU admission was 39.8 (SD 17.7) points. Renal replacement therapy was associated with approximately twice the observed mortality. Severity of illness remained a major determinant of mortality after removing age and urine volume from SAPS 2. RRT use was similar up to 70 years but declined thereafter. Creatinine increased with age and BMI, while urine volume progressively declined with age. The reason for ICU dependency was associated with mortality, RRT use, creatinine, and urine volume. **Conclusions:** RRT, age and severity of illness were strongly associated with mortality in this large ICU cohort. The reason for ICU dependency, reflecting the underlying disease, was substantially associated with mortality, RRT use, creatinine, and urine volume.

## 1. Introduction

Serum creatinine, urine volume, and renal replacement therapy (RRT) are markers of renal impairment in critically ill patients [1]. In critically ill patients, RRT was associated with higher mortality. Yet RRT is a life-saving therapy for renal failure [2]. The association between mortality and RRT may therefore reflect both renal impairment and its treatment. Large studies have described prevalence of RRT and associated mortality without including ICU- and patient-level factors [2,3] or the underlying disease leading to ICU admission [4]. Smaller studies focused on BMI and did not include ICU- and patient-level factors [5,6,7]. Previous studies investigating the association between renal impairment and mortality have lacked information on creatinine and urine volume [2,4,5,6]. Previous studies were often restricted to selected renal subgroups, introducing post-selection and limiting insight into how the observed range of renal impairment markers relates to mortality [4,5,6,7,8]. 

A comprehensive assessment of kidney function in the ICU requires more than RRT status. Creatinine levels and urine volume provide complementary information on renal impairment, as RRT may also be initiated for refractory fluid overload only [9]. This is the first multicentre ICU cohort analysis that jointly considers creatinine, urine volume and RRT and integrates ICU- and patient-level factors to assess the associations of RRT and other covariables with mortality within the same multivariable model [2,3,4,5,6,7,8]. 

Therefore, our goal was to quantify the association between mortality and renal impairment markers in a large worldwide cohort from the nutritionDay ICU project over 2007 to 2024, incorporating creatinine levels, urine volume, and RRT, and accounting for ICU- and patient-level factors. We hypothesised that mortality would be related to both renal impairment markers and a range of ICU- and patient-level factors.

## 2. Methods

### 2.1. Study Design and Population

**Figure 1:**
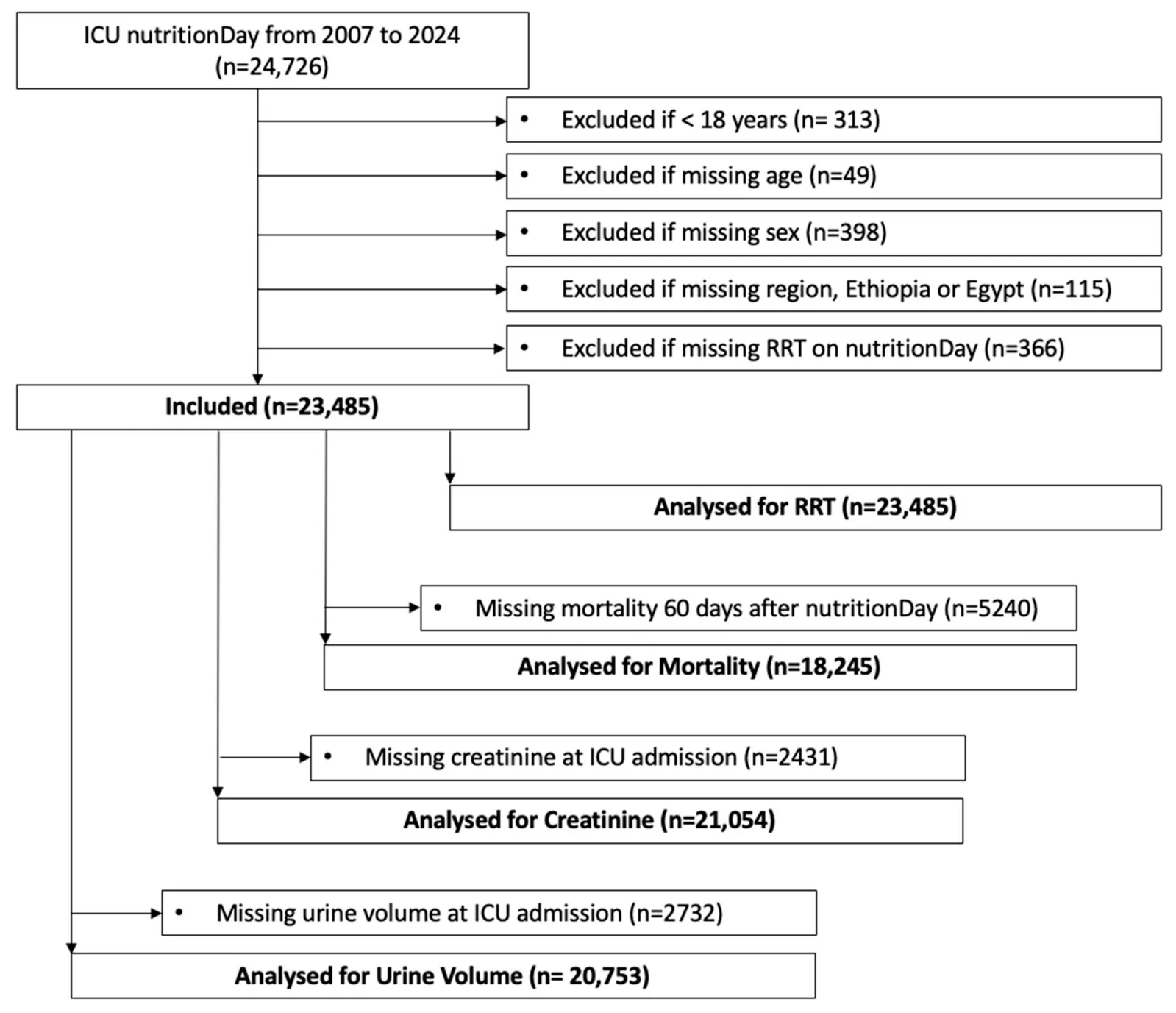
Study flowchart.

Briefly, nutritionDay in the ICU is an annual worldwide audit in which participating ICU health care providers prospectively record information on all ICU patients on a designated study day, which is called nutritionDay [10,11,12]. The nutritionDay project is a yearly repeated cross-sectional point-prevalence cohort study with 60-day outcome follow-up. This cross-sectional study design on a maximum of three possible inclusion days (first week: Thursday, second week: Wednesday, third week: Tuesday) in November captures the ICU patient population on that day. Each patient is recruited only once. nutritionDay therefore served as the study landmark and inclusion time. On nutritionDay, we assessed reasons for ICU dependency at admission, laboratory values obtained at ICU admission and on nutritionDay, and RRT use on nutritionDay. The nutritionDay questionnaire does not distinguish between intermittent and continuous RRT, or between acute and chronic RRT. Furthermore, the questionnaire does not assess indication, initiation time, duration, dose, net ultrafiltration, previous discontinuation, or subsequent initiation of RRT. Sixty days after nutritionDay, mortality, ICU and hospital length of stay were assessed. The ICU nutritionDay questionnaire used between 2007 and 2024, including all assessed variables, is provided in the Supplementary Materials. The participating ICUs were in Europe (50%), North America (6%), Latin America (18%) and Asia (25%) (Table S1, Supplementary Materials). In the present post hoc analysis, all adult ICU patients who participated between 2007 and 2024 were included. We excluded patients younger than 18 years (*n* = 313) or with missing ICU- or patient-level factors essential to the analysis, such as age (*n* = 49), sex (*n* = 398) or region (*n* = 88). Exclusions were applied sequentially, with each patient assigned to only one exclusion category; thus, the exclusion categories were mutually exclusive. Missing values denote variables not documented on the study day. African regions (Ethiopia (*n* = 26), Egypt (*n* = 1)) were excluded because of the small African sample size (Figure 1). Missing values for outcomes are indicated in Figure 1. The nutritionDay project was approved by the Ethics Committee of the Medical University of Vienna (EK1080/2020). Additional ethical clearance was obtained from local or national committees in the participating countries as required. The nutritionDay project is registered at ClinicalTrials.gov (NCT02820246). During the preparation of this work, the authors used ChatGPT version 1.2026.048 to assist with looking up specific R (version 4.3.3) codes, troubleshooting R errors and improving the formulation of selected sentences.

### 2.2. Outcomes

The primary outcomes were 60-day hospital mortality after nutritionDay and renal replacement therapy (RRT) on nutritionDay. Because mortality was analysed as a binary 60-day hospital outcome rather than as a time-to-event outcome, no additional censoring rules were applied. RRT use included both continuous and intermittent RRT on nutritionDay. Additional outcomes were serum creatinine and urine volume as renal impairment markers at ICU admission. These additional outcomes were chosen to identify ICU- and patient-level factors associated with renal impairment markers already at ICU admission, rather than only on nutritionDay or 60 days later.

### 2.3. Covariables Considered for Analyses

Covariables included ICU- and patient-level factors. The covariables included in the models were selected a priori as the clinically most relevant factors with a plausible temporal relationship to mortality and RRT. Missing values for independent covariables were retained and reported as a category for each covariable, because missingness may be informative [11]. Independent continuous covariables were categorised to provide clinically easily interpretable estimates without assuming a linear association and to allow inclusion of missing-value categories. Categories were specified before fitting the models and were not selected according to observed outcome associations. Established clinical categories were used where available, such as WHO BMI categories. Otherwise, quantile-based categories were chosen to ensure adequately sized groups and to permit an interpretable description across the observed distribution. Although categorisation may reduce statistical information, it was considered appropriate for this descriptive exploratory analysis intended to characterise clinically interpretable associations of a broad range of patient- and ICU-level variables with mortality and renal outcomes.

ICU-level factors included world region, with Europe as the reference group. World regions were defined according to the definition of the United Nations [13]. Australia was included in the Asia region for operational reasons. Calendar year periods were divided into half decades, with 2007–2011 as the reference group. ICU days, defined as time from ICU admission to the data collection on nutritionDay, were categorised into 0 to 1 days (*n*: 5373), 2 to 3 days (*n*: 4427), 4 to 6 days (reference group; *n*: 3078), 7–14 days (*n*: 3794), 15 or more days (*n*: 4688) and missing (*n*: 2125). Patient-level factors included sex, with female sex as the reference group, and age, categorised into 18–30 years (*n*: 1415), 30–40 years (*n*: 1600), 40–50 years (*n*: 2326), 50–60 years (*n*: 3956), 60–70 years (reference group; *n*: 5569), 70–80 years (*n*: 5549), 80–90 years (*n*: 2715) and 90 or more years (*n*: 355). BMI was categorised into the WHO categories [10–18.5) kg/m^2^ (*n*: 1072), [18.5–25) kg/m^2^ (*n*: 9089) as reference group, [25–30) kg/m^2^ (*n*: 7424), [30–35) kg/m^2^ (*n*: 2963), [35–40) kg/m^2^ (*n*: 1122), [40 and more) kg/m^2^ (*n*: 806) and missing BMI (*n*: 1009). Serum creatinine at ICU admission was recorded as the maximum value on the day of ICU admission. Urine volume at ICU admission was recorded as 24 h urine volume measured by urometry. Urine volume (mL/24 h) and BMI (kg/m^2^) were analysed separately to assess their independent associations and to avoid mathematical coupling introduced by weight-normalising urine volume while simultaneously including BMI. Serum creatinine and urine volume at ICU admission were categorised into quintiles instead of dichotomised into acute kidney injury (yes/no), allowing associations across the observed range to be assessed. For creatinine and urine volume, the middle quintile was defined as the reference group, corresponding to creatinine 0.9–1.2 mg/dL and urine volume 1200–1700 mL. The adjusted Simplified Acute Physiology 2 Score (SAPS 2) at ICU admission was calculated by excluding age and urine volume to avoid collinearity between covariables in the multivariable model. The adjusted SAPS 2 score was categorised into quintiles with the middle category as a reference, corresponding to an adjusted SAPS 2 of 19–26 points. Finally, patient-level factors included the main reasons for ICU dependency at ICU admission, categorised as abdominal, burn, cardiac, neurologic, pulmonary, septic, trauma and other ICU admissions, each coded as yes/no with “no” as the reference category. Reasons for ICU dependencies were multiple-response binary variables, and patients could therefore belong to more than one category. Each reason for ICU dependency was included as a separate binary covariable in the multivariable model and interpreted while adjusting for the other reasons.

### 2.4. Statistical Analysis

Continuous variables were described as the mean ± SD and/or median (Q1–Q3). Mean ± SD were retained because creatinine and urine volume were subsequently analysed using linear models. Covariables and outcomes were described across world regions. Pairwise associations between covariables were assessed by Kendall’s Tau test. Covariables were categorised to avoid assumptions about the functional form of association with the target variables, provide readily interpretable estimates, and allow inclusion of missing-value categories [11]. Reference categories correspond to an odds ratio of 1 in logistic models and a mean deviation of 0 in linear models. Reference groups for world regions were Europe, for calendar years the period 2007–2011, for time from ICU admission to data collection on nutritionDay 4–6 ICU days, female sex, age of 60–70 years, BMI 18.5–25 kg/m^2^, no RRT, creatinine middle quintile (0.9–1.2 mg/dL), urine volume 1200–1700 mL and adjusted SAPS 2 middle quintile (19–26 points).

Sixty-day hospital mortality was primarily assessed in a multivariable mixed-effects logistic regression, including all covariables and RRT as fixed effects, using the “meglm” command in Stata version 18.0. Individual ICUs were included as a random factor to account for clustering of patients within ICUs (Stata code in Figure S2, Supplementary Materials). No additional country-level random effect was specified, as the number of patients per country was highly variable, making a stable additional country-level variance estimate difficult (ExcelFile1_sup_country_year_patient, Supplementary Materials). As noted above, world region was already included as a fixed-effect covariable. To assess whether the association between RRT and mortality varied by reason for ICU dependency, we performed a global likelihood ratio test of the RRT-by-ICU-dependency interaction terms.

In a sensitivity analysis, we repeated the mortality model with adjustment for ICU length of stay using two approaches. The target population comprised adult ICU patients represented by the nutritionDay cross-sectional sample, with nutritionDay as the sampling landmark, sixty-day hospital mortality as the outcome, and the same covariables as in the primary mortality model. First, we applied the length-of-stay weighting algorithm for cross-sectional studies described by De Uña-Álvarez, which corrects for the greater probability of cross-sectional inclusion among patients with longer stays by assigning greater weight to patients with shorter stays [14,15]. In a second pragmatic approach, patients were weighted by 1/ICU length of stay, and values of 0 days were set to 1 day ICU length of stay. Both sets of patient-level probability weights were applied to the complete multivariable mixed-effects logistic regression, retaining ICU as a random effect (R and Stata code in Figure S1, Supplementary Materials).

Patients with and without available mortality data were descriptively compared. Sensitivity analyses were performed either with complete cases excluding patients with missing values for independent covariables or with multiple imputation of both missing values for independent covariables and outcome variables (Stata code in Figure S2, Supplementary Materials). Missing data were handled using multiple imputation by chained equations under a missing-at-random assumption conditional on the variables included in the imputation model, generating 25 imputed datasets. Binary mortality was imputed using logistic regression, while categorical variables with missing values were imputed using multinomial logistic regression. Each imputed dataset was subsequently analysed using the prespecified multilevel logistic regression model including a random intercept for ICU, and estimates were pooled across imputations (Stata code in Figure S2, Supplementary Materials). In the complete-case sensitivity analyses, the adjusted SAPS 2 was considered missing if more than three components were unavailable. In the imputation sensitivity analyses, the adjusted SAPS 2 scores, classified as missing, were imputed.

As a further sensitivity analysis for missing 60-day hospital mortality, we performed an inverse-probability-weighted (IPW) analysis. The probability of mortality outcome availability was estimated using logistic regression based on calendar year, country, ICU type, number of recruited patients per ICU, number of missing components in the original SAPS 2 score, ICU days, sex, age, and reasons for ICU dependency. Among patients with available mortality data, the primary multivariable mixed-effects logistic regression was then repeated using inverse probabilities of outcome availability as weights (Stata code in Figure S3, Supplementary Materials).

In another sensitivity analysis, the primary multivariable mixed-effects logistic regression and the above-described multiple-imputation and complete-case sensitivity analyses were performed when including the original SAPS 2 instead of the adjusted SAPS 2. In the complete-case sensitivity analyses, the original SAPS 2 was considered missing if one or more components were unavailable. In the imputation sensitivity analyses, the original SAPS 2 scores, classified as missing, were imputed. Including the original SAPS 2 score may, however, introduce collinearity and mathematical coupling because both age and urine volume are components of the original SAPS 2 score and were also included as separate independent variables in the model.

In additional sensitivity analyses for mortality, creatinine, urine volume, and adjusted SAPS 2 were modelled as continuous variables using restricted cubic splines with five knots instead of categorical variables. The otherwise unchanged multivariable mixed-effects logistic regression model included all other covariables as fixed effects and the individual ICU as a random effect (Stata code in Figure S5, Supplementary Materials).

Similarly, use of RRT on nutritionDay was assessed primarily in a multivariable mixed-effects logistic regression and subsequent complete-case and imputation sensitivity analyses. Covariables were included as fixed effects, with the individual ICU included as a random effect, analogous to the mortality model.

To identify covariables associated with renal impairment markers at ICU admission, creatinine and urine volume at ICU admission were analysed primarily in multivariable mixed-effects linear models and subsequent complete-case and imputation sensitivity analyses. All covariables, except ICU days and RRT, were included as fixed effects, and the individual ICU was included as a random effect, using the “meglm” command in Stata 18.0. ICU days, defined as duration from ICU admission to nutritionDay, and RRT on nutritionDay were not included in models of renal impairment markers at ICU admission, as both refer to a subsequent time point. Baseline creatinine and urine volume in multivariable linear regressions represent the model-estimated means when all covariables are in their reference groups. Residual diagnostics of the latter models were performed on creatinine, log-transformed creatinine and urine volume. As log transformation of urine volume was not possible due to zero values in anuric patients, urine volume was categorised into below and above 500 mL and analysed in a multivariable mixed-effects logistic regression, with all covariables, except ICU days and RRT, included as fixed effects and the individual ICU included as a random effect.

Figures present estimates or odds ratios with their corresponding 95% confidence intervals (95% CI). Odds ratios in the figures are displayed on a logarithmic scale. No adjustment for multiplicity was applied, consistent with the descriptive nature of this study and precluding any interpretation of the findings as causal effects. Statistical analyses were performed in Stata version 18.0 or R version 4.3.3. Figures were created in Stata 18.0 or PowerPoint 16.

## 3. Results

### 3.1. Study Population

The study included 23,485 adult patients from 1138 ICU wards in 60 countries from 2007 to 2024. The repeated participation of ICUs resulted in a total of 1855 ICU-year units (Excel Sheets in Supplementary Materials). Mean age was 61.4 (SD 17.0) years. Mean BMI was 26.6 (SD 6.3) kg/m^2^ and ranged from 25.2 (SD 5.3) in Asia to 29.0 (SD 8.3) kg/m^2^ in North America (Table 1 and Table S1, Supplementary Materials). Forty percent were female. Mean SAPS 2 at ICU admission was 39.8 (SD 17.7) points and ranged from 35.4 (SD 15.7) in North America to 41.8 (SD 17.6) points in Europe. Median creatinine at ICU admission was 1.00 (Q1–Q3, 0.74–1.60) mg/dL and ranged from 0.98 (Q1–Q3, 0.70–1.52) mg/dL in Latin America to 1.16 (Q1–Q3, 0.80–1.90) mg/dL in North America. Median urine volume at ICU admission was 1430 (Q1–Q3, 800–2200) mL and ranged from 1155 (Q1–Q3, 600–1900) mL in Latin America to 1550 (Q1–Q3, 900–2370) mL in Europe. RRT frequency on nutritionDay was about 10% in all world regions. nutritionDay data collection occurred at a median (Q1–Q3) of 4 (1–13) days after ICU admission. About half of the ICU patients were mechanically ventilated on nutritionDay. Sixty-day hospital mortality was 24% and ranged from 16% in North America to 27% in Latin America (Table 1 and Table S1, Supplementary Materials). No substantial pairwise collinearities between covariables were found. Pairwise Kendall correlations were low, with a maximum correlation coefficient of 0.23 between creatinine at ICU admission and RRT, indicating no substantial pairwise associations. Independent covariables and baseline characteristics were similar between patients with and without available mortality data (Table S2, Supplementary Materials).

### 3.2. Sixty-Day Hospital Mortality

**Figure 2:**
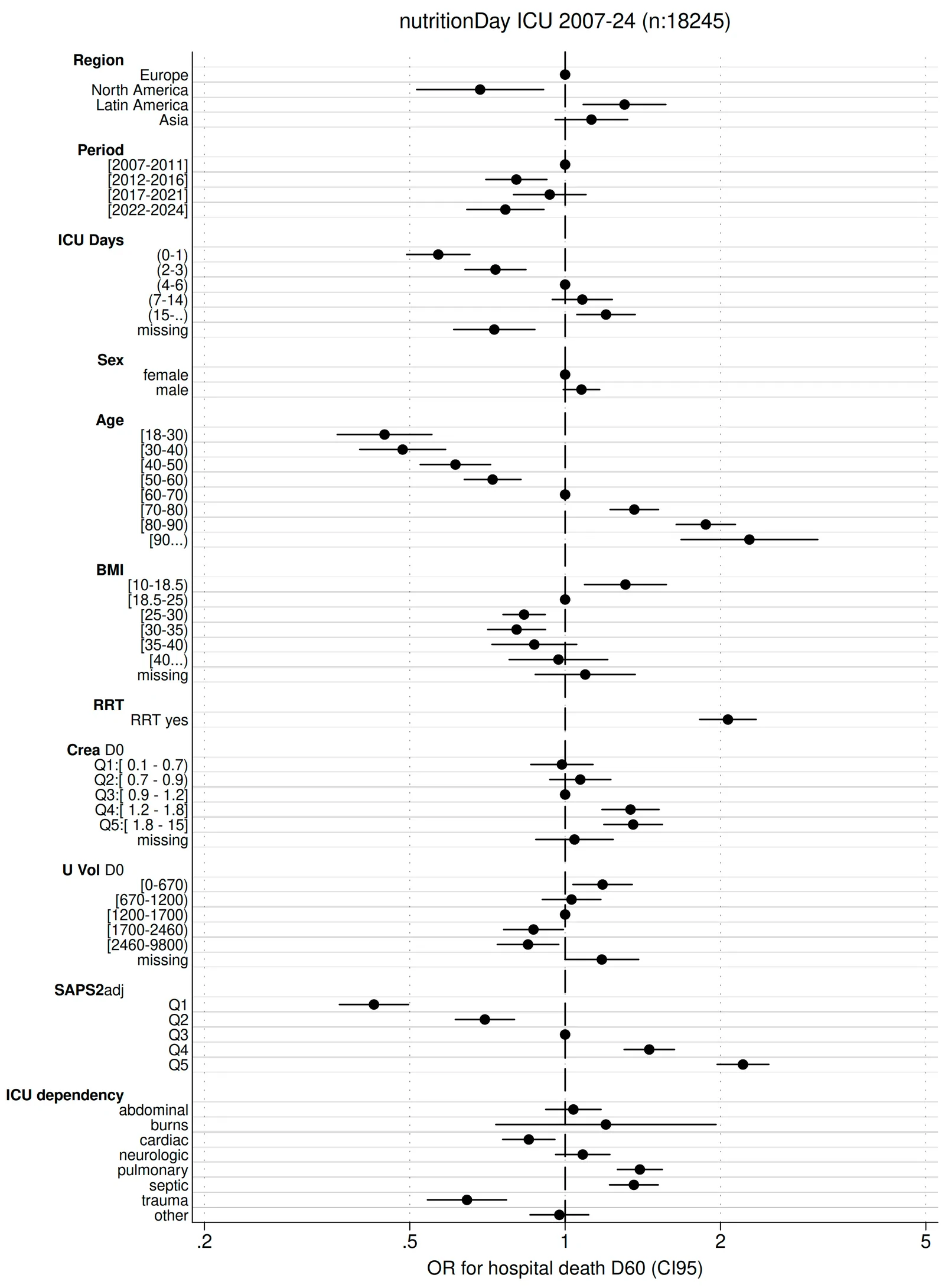
Association between 60-day hospital mortality and ICU- and patient-level factors in a multivariable mixed-effect logistic regression. Odds ratios (ORs) relative to the reference category (OR = 1) are presented with 95% confidence intervals. Hospital death D60: sixty-day hospital mortality; ICU Days in days; Age in years; BMI in kg/m^2^; RRT: Renal Replacement Therapy; Crea D0: creatinine at ICU admission in mg/dL; U Vol D0: urine volume at ICU admission in mL; SAPS 2adj: adjusted SAPS 2 score without age and urine volume components.

Renal replacement therapy was associated with approximately twice the observed mortality, 804/1823 (44%), compared with the reference group 3605/16,422 (22%). Patients in the lowest age and illness-severity categories had approximately fourfold lower mortality than those in the highest categories (Figure 2 and Table S3, Supplementary Materials). Severity of illness remained a major determinant of mortality, even after age and urine volume were removed from the original SAPS 2 score. The reason for ICU dependency had a substantial association with mortality. Pulmonary and septic ICU admissions were associated with higher mortality odds, whereas cardiac and trauma ICU admissions were associated with lower odds. Mortality was higher among patients who had already spent longer in the ICU by nutritionDay. BMI showed a U-shaped relationship with mortality, with lowest odds for BMI 25–35 kg/m^2^. Mortality was higher with creatinine above 1.2 mg/dL at ICU admission and increased only slightly in the lowest quintile of urinary volume at ICU admission. North America and the calendar years 2012–2016 and 2022–2024 were associated with lower mortality odds (Figure 2 and Table S3, Supplementary Materials).

The overall interaction between RRT and ICU dependency was not statistically significant in the mortality model (likelihood ratio test, χ^2^(8) = 13.98, *p* = 0.082). The associations with mortality remained unchanged after accounting for the cross-sectional design by weighting for ICU length of stay (Figure S1, Supplementary Materials). The associations with mortality were unchanged in complete-case analyses excluding patients with missing independent covariables and in analyses imputing missing independent covariables and mortality (Figure S2, Supplementary Materials). The associations with mortality remained unchanged with inverse probability weighting for mortality availability (Figure S3, Supplementary Materials). When including the original SAPS 2 instead of the adjusted SAPS 2, calculated without age and urine volume, in the multivariable analysis, associations of both age and urine volume with mortality were altered due to collinearity, as age and urine volume are already accounted for in the original SAPS 2 (Figure S4, Supplementary Materials vs. Figure 2). The associations with mortality were similar whether creatinine, urine volume, and adjusted SAPS 2 were modelled as continuous variables using restricted cubic splines or as categorical variables (Figures S5–S7, Supplementary Materials).

### 3.3. Renal Replacement Therapy on NutritionDay

**Figure 3:**
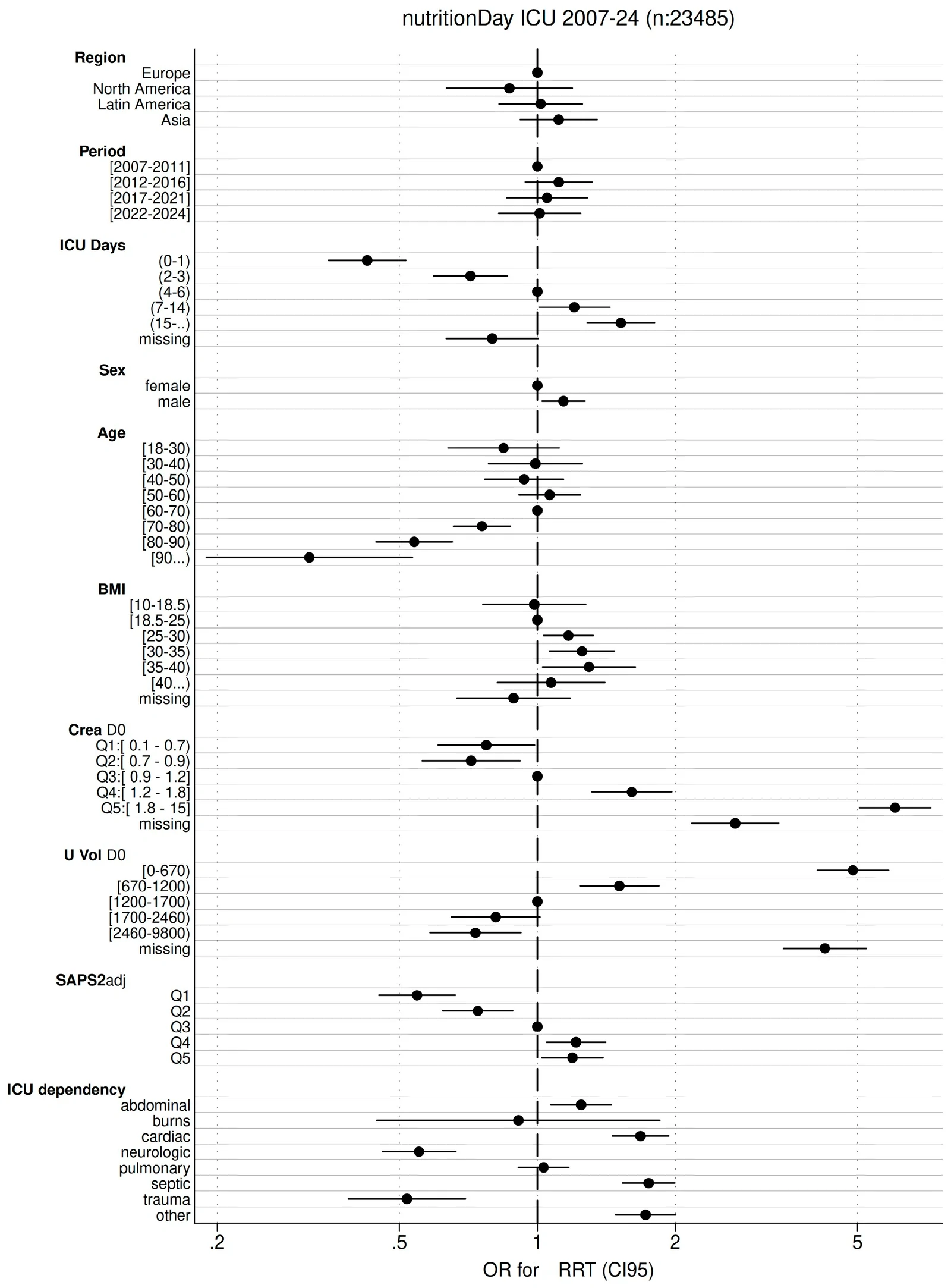
Associations between renal replacement therapy (RRT) and ICU- and patient-level factors in a multivariable mixed-effects logistic regression. Odds ratios (OR) relative to the reference category (OR = 1) are presented with 95% confidence intervals. ICU Days in days; Age in years; BMI in kg/m^2^; Crea D0: creatinine at ICU admission in mg/dL; U Vol D0: urine volume at ICU admission in mL; SAPS 2adj: adjusted SAPS 2 score without age and urine volume components.

RRT use was similar for all age categories up to 70 years of age but decreased thereafter (Figure 3 and Table S4, Supplementary Materials). The reason for ICU dependency had a substantial association with RRT. Abdominal, cardiac, and septic ICU admissions were associated with higher odds of RRT, whereas neurologic and trauma ICU admissions were associated with lower odds. Men had slightly higher odds for RRT than women. BMI showed an inverse U-shaped relationship with RRT, with higher odds for BMI 25–40 kg/m^2^. Odds of RRT increased with higher creatinine and lower urine volume at ICU admission. Use of RRT was similar across world regions and stable over calendar years (Figure 3 and Table S4, Supplementary Materials). The associations with RRT were unchanged in complete-case analyses excluding patients with missing independent covariables and in analyses imputing missing independent covariables (Figure S8, Supplementary Materials).

### 3.4. Creatinine at ICU Admission

**Figure 4:**
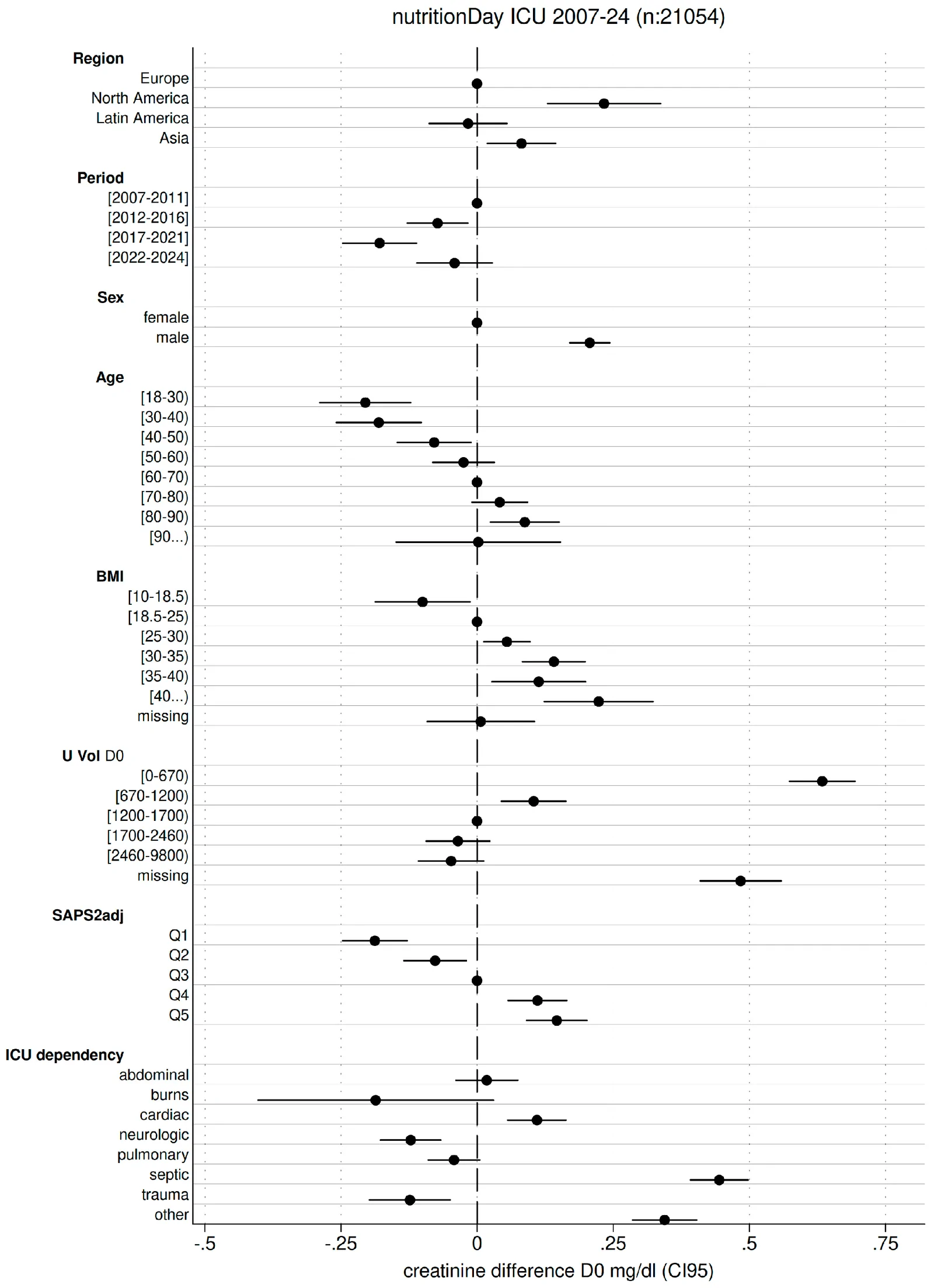
Mean deviations of serum creatinine at ICU admission (D0) from baseline creatinine for ICU- and patient-level factors in a multivariable mixed-effects linear regression. Mean deviations are presented with 95% confidence intervals. Baseline creatinine, expressed as the mean [95% CI] when all covariables are in their reference groups, was 1.07 [0.98–1.17] mg/dL. Age in years; BMI in kg/m^2^; U Vol D0: urine volume at ICU admission in mL; SAPS 2adj: adjusted SAPS 2 score without age and urine volume components.

Creatinine steadily increased with higher age and BMI (Figure 4 and Table S5, Supplementary Materials). Creatinine was higher in male than in female patients. The reason for ICU dependency was substantially associated with creatinine. Septic ICU admissions showed the highest creatinine levels, followed by cardiac ICU admissions, whereas creatinine levels were lower in neurologic and trauma ICU admissions. Creatinine increased with lower urine volume, particularly in the lowest urine volume quintile.

Severity of illness was still associated with higher creatinine, even after age and urine volume were removed from the original SAPS 2 score. Creatinine was higher in North America and Asia and lower in the calendar years 2012–2021 (Figure 4 and Table S5, Supplementary Materials). The associations with creatinine were unchanged in complete-case analyses excluding patients with missing independent covariables and in analyses imputing missing independent covariables and creatinine (Figure S9, Supplementary Materials). Residual diagnostics for creatinine indicated heteroscedasticity. Log transformation of creatinine improved residual behaviour, while the direction and pattern of associations remained unchanged.

### 3.5. Urine Volume at ICU Admission

**Figure 5:**
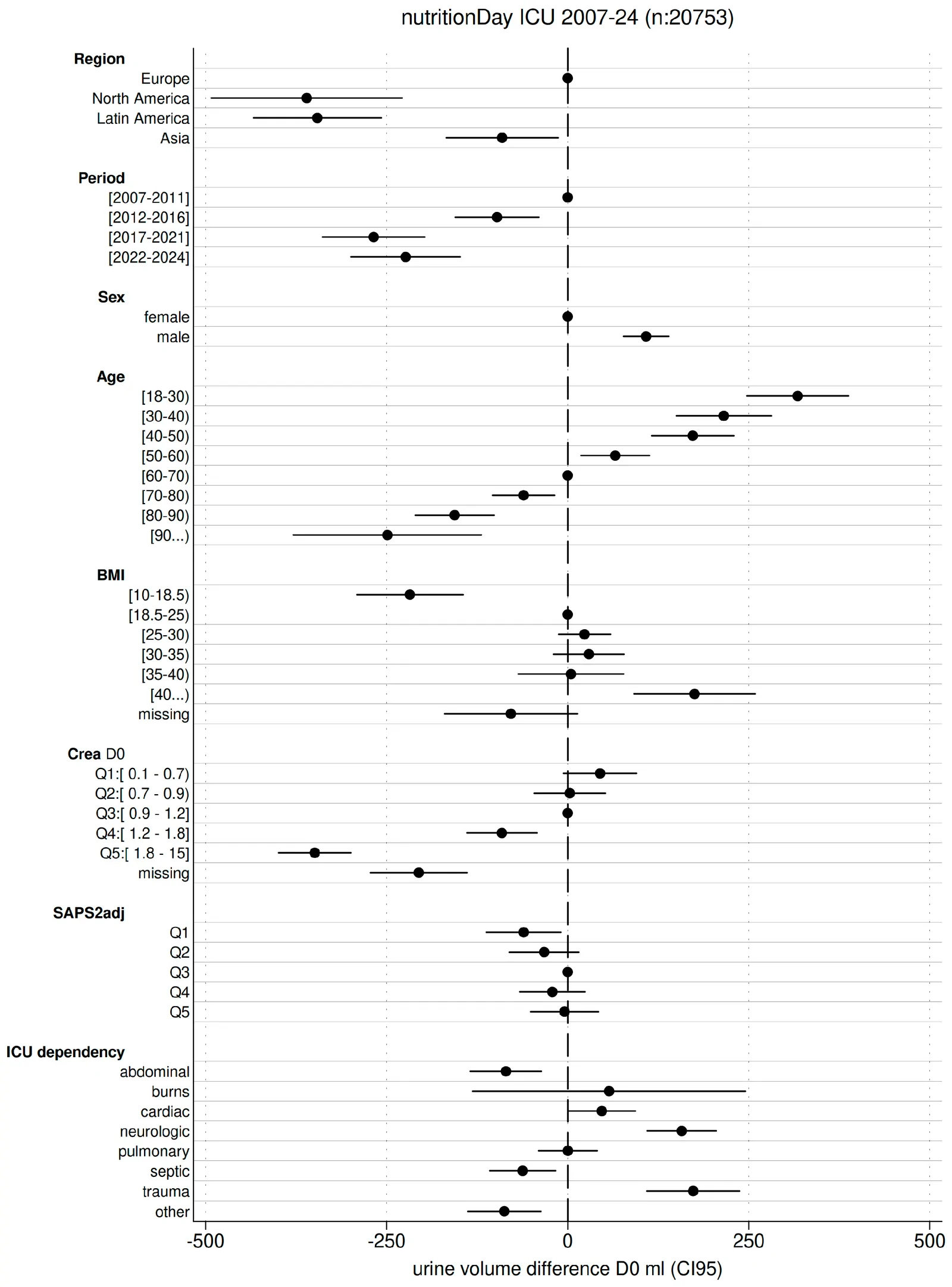
Mean deviations of urine volume at ICU admission (D0) from baseline urine volume for ICU- and patient-level factors in a multivariable mixed-effects linear regression. Mean deviations are presented with 95% confidence intervals. Baseline urine volume, expressed as the mean [95% CI] when all covariables are in their reference groups, was 1872 [1784–1960] mL. Age in years; BMI in kg/m^2^; Crea D0: creatinine at ICU admission in mg/dL; SAPS 2adj: adjusted SAPS 2 score without age and urine volume components.

Urine volume at ICU admission showed a pronounced decrease with age (Figure 5 and Table S6, Supplementary Materials). Urine volume was lowest and highest at the extremes of BMI (Figure 5). Men had higher urine volume than women. Urine volume decreased with a creatinine ≥ 1.2 mg/dL. The reason for ICU dependency was substantially associated with urine volume. Neurologic, trauma and cardiac ICU admissions showed higher urine volume, whereas abdominal and septic ICU admission showed lower urine volume. Urine volume was lower in all other world regions compared to Europe and lower from 2012–2024 compared to 2007–2011 (Figure 5 and Table S6, Supplementary Materials). The associations with urine volume were unchanged in complete-case analyses excluding patients with missing independent covariables and in analyses imputing missing independent covariables and urine volume (Figure S10, Supplementary Materials). Residual diagnostics for urine volume indicated heteroscedasticity. The OR for higher vs. lower urine volume (≥500 vs. <500 mL) across ICU- and patient-level factors remained similar in a multivariable mixed-effects logistic regression (Figure S11, Supplementary Materials).

## 4. Discussion

Renal replacement therapy, age, and severity of illness were prominently associated with mortality in this large multinational cohort study including ICU- and patient-level factors. The reason for ICU dependency, reflecting the underlying disease, substantially influenced mortality, RRT use, creatinine, and urine volume.

RRT may reflect both organ failure and its treatment. On the one hand, RRT was associated with higher mortality, supporting its role as a marker of severe organ dysfunction. On the other hand, the decision to initiate RRT is influenced by multiple factors beyond renal impairment, including treatment limitations, expected prognosis, local practice patterns, and clinician preference. Thus, the observed association between RRT and mortality is likely affected by confounding by indication. Because the timing of RRT initiation was unavailable, the temporal relationship between RRT and concurrent illness severity- and treatment-related variables assessed on nutritionDay could not be established. Residual confounding by current illness severity and other ICU therapies therefore remains possible. 

In our study, RRT was used less frequently in patients older than 70 years despite their higher mortality. This finding may reflect treatment selection, although higher creatinine and lower urine volume with increasing age were consistent with greater kidney dysfunction in older patients. Yet treatment decisions were not directly assessed, precluding causal inference. In contrast, use of RRT was most frequent in ICU patients above 80 years in a retrospective secondary analysis of German ICUs despite this group having the lowest ICU admission rate [3]. These differences may reflect country-specific practice patterns, differences in patient selection, and the retrospective design of that study [3].

In contrast to previous studies [2,4,5,6,7], we comprehensively integrated all ICU- and patient-level factors to assess the relationship between mortality and renal impairment markers. The association between mortality and renal impairment markers is multifactorial and involves ICU-, patient-level factors, treatment decisions, and potential biases. First, the underlying disease responsible for ICU admission was substantially associated with RRT use and mortality. Cardiac ICU admissions were more likely to receive RRT but had lower mortality. Septic ICU admissions had higher odds of RRT and mortality. Neurologic ICU admissions were less likely to receive RRT but had higher mortality. Second, the apparent contradiction of higher RRT and lower mortality for BMI 25–35 kg/m^2^ resembles the obesity paradox [16]. The obesity paradox may be explained by both bias and biology. Proposed sources of bias in the previous literature include reverse causation due to disease-related weight loss; residual confounding due to incomplete control of confounders; treatment bias with obese patients more likely to be admitted to the ICU and receiving less body-weight adjusted fluids, vasopressors and nutrition; and publication bias favouring studies only reporting the obesity paradox [10,16,17,18,19]. Biological explanations in the previous literature include greater energy and muscle reserves to withstand critical illness, potential immunomodulating qualities of adipose tissue and a different gut microbiota [5,6,16,20,21]. These explanations remain hypotheses in the context of our data.

Moreover, sex, age and BMI were patient-level factors associated with renal impairment markers. On the one hand, the higher creatinine levels with increasing BMI may reflect greater muscle mass [22,23]. On the other hand, a higher BMI is known to be associated with glomerular hyperfiltration [24,25,26], which may explain the observed higher urine volume. Over time, glomerular hyperfiltration may lead to a decline in the glomerular filtration rate [26], which may partly explain the higher RRT use with increasing BMI in our study. Interestingly, age showed a particularly pronounced association with urine volume, which progressively decreased with increasing age. A decrease in estimated GFR, but not in urine volume itself, has been shown previously with higher age [27,28]. To our knowledge, our results show for the first time a progressive decrease in daily urine volume with age. In men, higher creatinine levels and urine volumes may partly reflect greater muscle and body mass [29]. This may have contributed to the higher odds of RRT observed in men without a corresponding increase in mortality compared with women. These observations suggest that differences in body composition related to sex, age and BMI may contribute to differences in renal impairment markers and RRT use.

Finally, ICU-level factors, such as world region and calendar year, were also associated with mortality and should therefore always be reported. Among world regions, patients in North America had the highest creatinine, with lower urine volume, but tended to receive RRT less frequently while still showing the lowest hospital mortality. This apparent contradiction may partly reflect system-inherent factors like an early transfer to secondary health care institutions in North America, patient selection and differing thresholds for initiating RRT. Because participation of countries and ICUs varied across world regions and calendar periods, these geographic and temporal comparisons are conditional on the participating units and should be interpreted descriptively rather than as population-representative regional or temporal effects. Patients treated during 2017 to 2021 showed the lowest creatinine and urine volume, similar odds for RRT and a trend toward higher odds of mortality. The latter may partly reflect the COVID-19 pandemic, which overlapped with the 2017–2021 period. Both urine volume and creatinine were lower after 2012 than before 2012, potentially reflecting a more fluid-restrictive approach over time in intensive care. In contrast to a German retrospective study [3], but similar to another large international retrospective study [2], we found no increase in RRT use over the study period in our multinational prospective study. All of the above interpretations remain hypotheses in the context of our observational study.

The main strength of our study was the first-time comprehensive assessment of renal impairment markers and integration of all ICU- and patient-level factors in a large worldwide sample size of ICU patients with different underlying diseases. Moreover, previous ICU studies were restricted to patients with renal impairment, introducing post-selection and limiting insight into how the observed range of renal impairment markers relates to mortality [5,6,7]. In contrast, we included patients across the full range of observed renal impairment markers. This allowed us to evaluate the independent associations of RRT and other covariables with mortality across the full ICU cohort.

We acknowledge that our convenience sample, derived from a global initiative primarily designed to improve nutritional care and raise awareness of disease-related malnutrition, had inherent limitations regarding population representativeness, range of variables collected, and completeness of documentation. For example, the nutritionDay questionnaire did not allow us to distinguish between intermittent and continuous RRT or between acute and chronic renal impairment, potentially resulting in RRT exposure misclassification. Moreover, categorisation of variables may entail some loss of information and residual confounding. Yet our descriptive analysis was not intended to estimate causal exposure–response relationships, and we therefore retained categorised variables to explore clinically interpretable associations without imposing a specific functional form on potentially nonlinear relationships. Moreover, the associations with mortality were similar whether creatinine, urine volume, and adjusted SAPS 2 were modelled as continuous variables using restricted cubic splines or as categorical variables. Another relevant limitation was the study’s cross-sectional design. The cross-sectional design warrants cautious interpretation, as findings indicate associations rather than causal relationships. In cross-sectional studies, long-stay patients may be overrepresented. However, we accounted for the length-of-stay bias inherent to the cross-sectional design and found that associations between renal impairment markers and mortality remained unchanged. A cross-sectional design may also offer some advantages, as it includes a higher proportion of severely ill patients than a cohort study, thereby allowing more precise estimates in high-risk patients while reducing the data collection workload. Another limitation was missing data. However, we accounted for missingness by reporting it as a category for each covariable, because missingness may be informative [11]. Mortality was missing in 22% of included patients. Yet complete-case, multiple-imputation and inverse-probability-weighted sensitivity analyses yielded similar results, reinforcing the consistency of the observed associations.

## 5. Conclusions

Age, adjusted SAPS 2 score and RRT were strongly associated with sixty-day hospital mortality of ICU patients in this large worldwide cohort. The reason for ICU dependency, reflecting the underlying disease, was substantially associated with mortality, RRT use, creatinine, and urine volume. Sex-, age- and BMI-related differences in body stature and composition may partly reflect differences in renal impairment markers and RRT use.

## Figures and Tables

**Table 1:** Baseline characteristics, renal impairment markers and mortality.

*n*	23,485
**ICU-Level Factors**	
World Region (in *n* = 23,485)	
Europe (*n*)	11,837 (50%)
North America (*n*)	1477 (6%)
Latin America (*n*)	4264 (18%)
Asia (*n*)	5907 (25%)
Time Period (in *n* = 23,485)	
2007–2011	6224 (27%)
2012–2016	7657 (33%)
2017–2021	4471 (19%)
2022–2024	5133 (22%)
Time from ICU admission to nutritionDay, termed “ICU days” Mean (SD) Median (Q1–Q3) (in *n* = 21,360)	11.5 (SD 21.3) 4 (1–13)
**Patient-Level Factors at ICU Admission**	
Female (*n*) (in *n* = 23,485)	9459 (40%)
Age (years) (in *n* = 23,485)	61.4 (SD 17.0)
BMI (kg/m^2^) (in *n* = 22,476)	26.6 (SD 6.3)
Creatinine at ICU admission (mg/dL) Mean (SD) Median (Q1–Q3) (in *n* = 21,054)	1.47 (SD 1.37) 1.00 (0.74–1.60)
BUN at ICU admission (mg/dL) Mean (SD) Median (Q1–Q3) (in *n* = 5167)	33.7 (SD 30.9) 23.0 (14.0–42.0)
Urine volume in 24 h at ICU admission (mL) Mean (SD) Median (Q1–Q3) (in *n* = 20,753)	1641.87 (SD 1181.70) 1430.00 (800.00–2200.00)
SAPS 2 score at ICU admission (points) (in *n* = 23,485)	39.8 (SD 17.7)
Adjusted SAPS 2 score at ICU admission without age and urine volume (points) (in *n* = 23,485)	25.8 (SD 15.4)
Reason for ICU dependency at ICU admission (*n*) (multiple reasons possible)	
Abdominal	3878 (17%)
Burns	174 (0.7%)
Cardiac	5199 (22%)
Neurologic	4370 (19%)
Pulmonary	6276 (27%)
Sepsis	3583 (15%)
Trauma	1927 (8%)
Other	3474 (15%)
**Variables On nutritionDay**	
Renal Replacement Therapy on nutritionDay (*n*) (in *n* = 23,485)	2313 (10%)
Hemofiltration	319 (1%)
Hemodialysis	1502 (6%)
Hemodiafiltration	492 (2%)
SOFA score at nutritionDay (points) (in *n* = 23,485)	3.8 (SD 3.3)
Creatinine at nutritionDay (mg/dL) (in *n* = 16,837) Mean (SD) Median (Q1–Q3)	1.34 (SD 1.23) 0.93 (0.66–1.50)
Urine volume in 24 h at nutritionDay (mL) Mean (SD) Median (Q1–Q3) (in *n* = 21,521)	1969.64 (SD 1328.40) 1800.00 (1055.00–2690.00)
No diuretics on nutritionDay (*n*) (in *n* = 23,485)	15,643 (67%)
Intermittent diuretics on nutritionDay (*n*) (in *n* = 23,485)	3366 (14%)
Continuous diuretics on nutritionDay (*n*) (in *n* = 23,485)	4476 (19%)
**Variables After nutritionDay**	
Time from ICU admission to hospital discharge (days), Mean (SD) Median (Q1–Q3) (in *n* = 16,781)	27.9 (SD 29.4) 18 (9–38)
60-day hospital mortality (*n*) (in *n* = 18,245)	4409 (24%)

BUN: Blood urea nitrogen.

## Data Availability

The raw data supporting the conclusions of this article will be made available by the authors on request.

## Supplementary Materials

The following supporting information can be downloaded at https://www.mdpi.com/article/10.3390/jcm15187119/s1: ExcelFile1_sup_country_year_patient.xlsx: Country-by-year participation tables for patients; ExcelFile2_sup_country_yearunit.xlsx: Country-by-year participation tables for ICU units; ExcelFile3_sup_repetitions_yearunit.xlsx: Number of ICU units × years of Participation; PdfFile1_sup_nday1_icu_en.pdf: nutritionDay ICU questionnaire 1; PdfFile2_sup_nday2_icu_en.pdf: nutritionDay ICU questionnaire 2; PdfFile3_sup_nday3_icu_en.pdf: nutritionDay ICU questionnaire 3; PdfFile4_sup_nday4_icu_en.pdf: nutritionDay ICU questionnaire 4; PdfFile5_sup_nday5_icu_en.pdf: nutritionDay ICU questionnaire 5. Supporting Information Word File WordFile_sup_inf.docx including below. Table S1. Baseline characteristics, renal impairment markers and mortality across different world regions; Table S2. Baseline characteristics and independent variables in patients with and without available mortality data; Table S3. Association between sixty-day hospital mortality and ICU- and patient-level factors in a multivariable logistic regression; Table S4. Associations between renal replacement therapy and ICU- and patient-level factors in a multivariable logistic regression; Table S5. Mean deviations of serum creatinine at ICU admission from baseline creatinine for ICU- and patient-level factors in a multivariable linear regression; Table S6. Mean deviations of urine volume at ICU admission from baseline urine volume for ICU- and patient-level factors in a multivariable linear regression; Figure S1. Sensitivity analysis for sixty-day hospital mortality accounting for the bias of ICU length of stay; Figure S2. Sensitivity analysis for sixty-day hospital mortality with complete cases excluding patients with missing values for independent covariables or with imputation of both missing values for independent covariables and mortality; Figure S3. Sensitivity analysis for sixty-day hospital mortality with inverse probability weighting for mortality availability or with imputation of both missing values for independent covariables and mortality; Figure S4. Sensitivity analysis including the original SAPS 2 score for sixty-day hospital mortality with complete cases excluding patients with missing values for independent covariables or with imputation of both missing values for independent covariables and mortality; Figure S5. Association between creatinine and sixty-day hospital mortality when using restricted cubic splines for creatinine, urine volume and adjusted SAPS 2 score; Figure S6. Association between urine volume and sixty-day hospital mortality when using restricted cubic splines for creatinine, urine volume and adjusted SAPS 2 score; Figure S7. Association between adjusted SAPS 2 score and sixty-day hospital mortality when using restricted cubic splines for creatinine, urine volume and adjusted SAPS 2 score; Figure S8. Sensitivity analysis for renal replacement therapy with complete cases excluding patients with missing values for independent covariables or with imputation of missing values for independent covariables; Figure S9. Sensitivity analysis for mean deviations of serum creatinine at ICU admission from baseline creatinine with complete cases excluding patients with missing values for independent covariables or with imputation of both missing values for independent covariables and creatinine; Figure S10. Sensitivity analysis for mean deviations of urine volume at ICU admission from baseline urine volume with complete cases excluding patients with missing values for independent covariables or with imputation of both missing values for independent covariables and urine volume; Figure S11. Sensitivity analysis for higher versus lower urine volume (≥500 vs. <500 mL) at ICU admission in a multivariable mixed-effects logistic regression.

## Abbreviations

BMI	Body mass index
BUN	Blood urea nitrogen
nDay	nutritionDay
RRT	Renal replacement therapy
ICU	Intensive care unit
SAPS 2 score	Simplified Acute Physiology 2 score
95% CI	95% confidence intervals
