# Supplementary material for: Mortality, Renal Replacement Therapy and Renal Impairment Markers in Critically Ill Patients in a Multinational Point-Prevalence ICU Cohort over 2007 to 2024: A nutritionDay Descriptive Multivariable Analysis
Source: J Clin Med. 2026 Sep 14;15(18):7119. doi: 10.3390/jcm15187119 (PMC13607511; doi:10.3390/jcm15187119)
Supplement: Supplementary file 1 [file jcm-15-07119-s001.zip › PdfFile1_sup_nday1_icu_en.pdf]

Please mark the correct boxes that apply to your unit

nutritionDay worldwide

INTENSIVE CARE UNIT SHEET 1

Date
Center-Code
Unit-Code

**Actual Number of ICU beds**	
**Maximum number of ICU beds**	

Type of ICU (please tick):

◯ Medical ICU ◯ surgical ICU ◯ interdisciplinary ICU ◯ HDU ◯ other

People working in your unit on nutritionDay:

	Number	
	**morning shift**	**night shift**
intensivist (> 75% on ICU)		
anesthesiologist		
internist		
pediatrician		
others		
nurses		
nursing aides		
Dieticians/ dietetic assistants		
physiotherapists		

**Is there a person on your unit dedicated to nutritional care?**	◯ YES	◯ No
**Is there a nutrition team in your hospital?**	◯ YES	◯ No
**Do you have written procedures for nutritional care?**	◯ YES	◯ No
**f YES, which one ...**		
ICU nutrition protocol	◯ YES	◯ No
national/international guidelines	◯ YES	◯ No
individual patient care plans	◯ YES	◯ No
**How do you assess the nutritional status of a patient?**		
clinical global assessment	◯ YES	◯ No
weight / height	◯ YES	◯ No
by checking laboratory parameters	◯ YES	◯ No
By using a score / questionnaire	◯ YES	◯ No
**Where do you prepare parenteral nutrition?**		
ICU	◯ YES	◯ No
pharmacy	◯ YES	◯ No
other	◯ YES	◯ No

Comment:

THANK YOU!
