# Supplementary material for: Mortality, Renal Replacement Therapy and Renal Impairment Markers in Critically Ill Patients in a Multinational Point-Prevalence ICU Cohort over 2007 to 2024: A nutritionDay Descriptive Multivariable Analysis
Source: J Clin Med. 2026 Sep 14;15(18):7119. doi: 10.3390/jcm15187119 (PMC13607511; doi:10.3390/jcm15187119)
Supplement: Supplementary file 1 [file jcm-15-07119-s001.zip › PdfFile2sup_nday2_icu_en.pdf]

Please mark the correct boxes that apply to the patient

nutritionDay worldwide

INTENSIVE CARE UNITS SHEET 2

Date ☐☐ ☐☐ ☐☐☐☐
Center-Code ☐☐☐☐☐
Unit-Code ☐☐☐☐☐

Patient number ☐☐☐ **Patient initials** [redacted] **Age** ☐☐☐ years

Sex ◯ Female ◯ Male

weight (on day of ICU-admission) ☐☐☐,☐ kg ◯ estimated ◯ measured *If postsurgery, please use preoperative weight*

height ☐☐☐ cm ◯ estimated ◯ measured

date of ICU admission ☐☐ ☐☐ ☐☐☐☐ *(dd/mm/yyyy)*

date of admission in hospital ☐☐ ☐☐ ☐☐☐☐ *(dd/mm/yyyy)*

Patient is ◯ medical ◯ surgical

OP is ◯ elective ◯ emergency number of days since OP ☐☐☐ days

main reasons for ICU dependency (at ICU admission)

☐ abdominal ☐ cardiac ☐ pulmonary ☐ trauma
☐ burns ☐ neurological ☐ septic ☐ others

Co-morbidities

☐ cancer therapy ☐ cirrhosis ☐ hematological cancer
☐ cancer, metastase ☐ heart failure (NYHA IV) ☐ AIDS

GLASGOW Coma Scale (admission Day)

Eyes	verbal	motor
◯ 1=Does not open eyes	◯ 1=Makes no sounds	◯ 1=Makes no movements
◯ 2=Opens eyes in response to painful stimuli	◯ 2=Incomprehensible sounds	◯ 2=Extension to painful stimuli (decerebrate response)
◯ 3=Opens eyes in response to voice	◯ 3=Utters incoherent words	◯ 3=Abnormal flexion to painful stimuli
◯ 4=Opens eyes spontaneously	◯ 4=Confused, disoriented	◯ 4=Flexion / Withdrawal to painful stimuli
	◯ 5=Oriented, converses normally	◯ 5=Localizes painful stimuli
		◯ 6=Obeys commands

LABORATORY (Admission day)

Parameter	UNIT	MIN	MAX	Parameter	UNIT	MIN	MAX
syst. blood pressure	mmHg			Potassium	◯ mEq/L ◯ mmol/L		
heart rate	bpm			Sodium	◯ mEq/L ◯ mmol/L		
Body temperature	◯ °C ◯ °F			pH			
PaO2/FiO2	mmHg			HCO3-	◯ mEq/L ◯ mmol/L		
urine output	ml (in 24h)			Bilirubin	◯ mg/dl ◯ µmol/l		
serum urea	◯ mg/dl ◯ mmol/L			Creatinine	◯ mg/dl ◯ µmol/l		
BUN	◯ mg/dl ◯ mmol/L			Glucose	◯ mg/dl ◯ mmol/L		
WBC	◯ 10^3/µL ◯ 10^9/L			Lactat	◯ mg/dl ◯ mmol/L		

THANK YOU!
