# Supplementary material for: Mortality, Renal Replacement Therapy and Renal Impairment Markers in Critically Ill Patients in a Multinational Point-Prevalence ICU Cohort over 2007 to 2024: A nutritionDay Descriptive Multivariable Analysis
Source: J Clin Med. 2026 Sep 14;15(18):7119. doi: 10.3390/jcm15187119 (PMC13607511; doi:10.3390/jcm15187119)
Supplement: Supplementary file 1 [file jcm-15-07119-s001.zip › PdfFile3sup_nday3_icu_en.pdf]

Please mark the correct boxes that apply to the patient

nutritionDay worldwide

INTENSIVE CARE UNITS SHEET 3

Date ☐☐ ☐☐ ☐☐☐☐
Center-Code ☐☐☐☐☐
Unit-Code ☐☐☐☐☐

Patient number ☐☐☐ **Patient Initials** [redacted]

TREATMENT (Actual day)

**sedated**	○ No	○ Intermittent	○ Continuous		
**paralyzed**	○ No	○ Intermittent	○ Continuous		
**GLASGOW Coma Scale** (Total score as if the patient would not be sedated)	☐				
**Sedation with propofol**	○ No	○ Yes	☐ ml/d		
**Ventilated (more than 8 hours)**	○ No	○ Intubated	○ Face mask	○ Helmet	○ Other
**Head position:**	○ 0	○ 30	○ 45	○ Other	
**Pain control**	☐ Opioids	☐ Epidural	☐ Other analgetics	☐ Other sedatives	☐ None
**Limitation of care**	○ No	○ Terminal Care	○ Limited Care		○ DNR
**Vasoactive support (>1 hour/day)**	☐ No	☐ Vasopressor	☐ Inotropics	☐ Vasodilator	
**Vasopressor dose**	○ Low Noradrenaline < 0.1 µg/kg/h		○ High Noradrenaline > 0.1 µg/kg/h		
**Insulin therapy**	○ No	○ Intermittent	○ Continuous	○ Pen	
**Antibiotic treatment**	○ Yes		○ No		
**Diuretics**	○ No	○ Intermittent	○ Continuous		
**Renal replacement therapy**	○ No	○ Hemofiltration	○ Hemodialysis	○ Hemodiafiltration	

LABORATORY (within 24 hours)

Parameter	UNIT	min	max
mean blood pressure	mmHg		
PaO2/FiO2	mmHg		
Urine output	ml (24h)		
Creatinine	○ mg/dl<br>○ µmol/l		
Glucose	○ mg/dl<br>○ mmol/L		
Bilirubin	○ mg/dl<br>○ µmol/l		
Lactat	○ mg/dl<br>○ mmol/L		
Platelets	G/l		

RAMSAY score (Actual day)

- ○ Anxious, agitated, restless
- ○ Cooperative, orientated, tranquil
- ○ Respond to commands only
- ○ Light glabellar tap => brisk response
- ○ Light glabellar tap => sluggish response
- ○ No response

NEMS (Actual Day)

1. ☐ Basic monitoring
2. ☐ Intravenous medication
3. ☐ Mechanical ventilatory support
4. ☐ Supplementary ventilatory care
5. ☐ Single vasoactive medication
6. ☐ Multiple vasoactive medication
7. ☐ Dialysis / Hemofiltration
8. ☐ Specific interventions in the ICU
9. ☐ Specific interventions outside the ICU

Please mark either 3 or 4, 5 or 6

Please refer to the explanation sheet for more infomation

INFECTIONS (within last 10 days)

Pneumonia	○ Yes	○ No	○ Not available
Urinary tract	○ Yes	○ No	○ Not available
Catheter	○ Yes	○ No	○ Not available
Wound	○ Yes	○ No	○ Not available

THANK YOU!

© nutritionDay - Authors: Hiesmayr / Schindler / Simon / Kiss / Kosak / Bauer - Medical University of Vienna / ESPEN
Version 1 - 07.10.2016
