# Supplementary material for: Mortality, Renal Replacement Therapy and Renal Impairment Markers in Critically Ill Patients in a Multinational Point-Prevalence ICU Cohort over 2007 to 2024: A nutritionDay Descriptive Multivariable Analysis
Source: J Clin Med. 2026 Sep 14;15(18):7119. doi: 10.3390/jcm15187119 (PMC13607511; doi:10.3390/jcm15187119)
Supplement: Supplementary file 1 [file jcm-15-07119-s001.zip › PdfFile4sup_nday4_icu_en.pdf]

Please mark the correct boxes that apply to the patient

nutritionDay worldwide

INTENSIVE CARE UNIT SHEET 4a

Date
Center-Code
Unit-Code

Patient number | **Patient Initials** [redacted]

NUTRITIONAL STATUS AND TREATMENT (Actual day)

Which lines and tubes does the patient have?

- Central venous
- Nasogastric
- Nasojejunal
- Percutaneous endosc. Gastrostomy
- Percutaneous endoscopy/surgical jejunostomy (PEJ)
- Periphervenous

Nutritional approaches

- Oral
- Enteral
- Parenteral
- No nutrition

Number of days of parenteral feeding on ICU days

Number of days of enteral feeding on ICU days

(= actual date - date of the first day ofparenteral or enteral nutrition given)

Duration of enteral nutrition (within the last 24 h) hours — Intermittent — Continuous

Reasons for interrupting nutritional support

- Surgery
- Transport
- Intolerance
- Other

Calories planned per kg for the next 24 hours kcal/kg/day

Or: <500 kcal/day — 500-999 kcal/day — 1000-1499 kcal/day — 1500-2000 kcal/day — >2000 kcal/day

Gastric reflux ml

Constipation or diarrhea Constipation > 3days — Diarrhea

Intra abdominal pressure measured No — Yes — mmHg

Is feeding orally possible? No — Yes

If yes, please tick: Drinking — Eating

If "NO", why not?

- Patient is sedated
- Not allowed to eat
- Cannot swallow
- Recent aspiration

If "YES", does the patient eat...

- Normal hospital food
- Supplements
- Just drinks

ORAL NUTRITION - Please indicate for one meal:

This meal was: **Lunch** — **Dinner**

about all — **1/2** — **1/4** — **nothing**

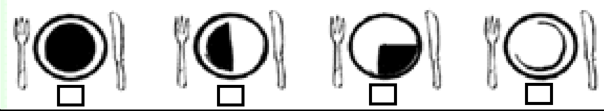

Ask your patient about feeling and wellbeing

1. Are you hungry?	Yes	No	Not available
2. Would you like to eat something?	Yes	No	Not available
3. Are you thirsty?	Yes	No	Not available
4. Do you have a dry mouth?	Yes	No	Not available
5. Do you feel nausea?	Yes	No	Not available
6. Do you have abdominal pain?	Yes	No	Not available

Please continue with sheet 4b

Please mark the correct boxes that apply to the patient

nutritionDay worldwide

INTENSIVE CARE UNIT SHEET 4b

Date ☐☐ ☐☐ ☐☐☐☐
Center-Code ☐☐☐☐☐
Unit-Code ☐☐☐☐☐

Patient number ☐☐☐ **Patient Initials** [redacted]

ENTERAL / PARENTERAL NUTRITION

Does the patient get an industrial finished product? ◯ YES ◯ No

ENTERAL nutrition product and volume [15]

Name: __________ CODE: ____

This product has	____	kcal/ml	***OR in kcal***	
Planned for the last 24 hours:	____	ml/24h	____	kcal/24h
Given within the last 24 hours:	____	ml/24h	____	kcal/24h

PARENTERAL nutrition product and volume

Name: __________ CODE: ____

This product has	____	kcal/ml	***OR in kcal***	
Planned for the last 24 hours:	____	ml/24h	____	kcal/24h
Given within the last 24 hours:	____	ml/24h	____	kcal/24h

Other nutrition product and volume

Name: __________ CODE: ____

This product has	____	kcal/ml	***OR in kcal***	
Planned for the last 24 hours:	____	ml/24h	____	kcal/24h
Given within the last 24 hours:	____	ml/24h	____	kcal/24h

Individualy composed products/additionals

if you use individually composed products, please fill in:

**AMINOACIDS:**	amount planned for the last 24 hours:	____	g/24h
	Amount given within the last 24 hours:	____	g/24h
**CARBOHYDRATES:**	amount planned for the last 24 hours:	____	g/24h
	Amount given within the last 24 hours:	____	g/24h
**LIPIDS:**	amount planned for the last 24 hours:	____	g/24h
	Amount given within the last 24 hours:	____	g/24h

Additional nutrients?

- ☐ Amino acids
- ☐ Glutamine
- ☐ MUFA
- ☐ Omega-3-fatty acids
- ☐ Glucose
- ☐ Vitamine E
- ☐ Selen

THANK YOU!

© nutritionDay - Authors: Hiesmayr / Schindler / Simon / Kiss / Kosak / Bauer - Medical University of Vienna / ESPEN Version 1 - 07.10.2016
