# Supplementary material for: Mortality, Renal Replacement Therapy and Renal Impairment Markers in Critically Ill Patients in a Multinational Point-Prevalence ICU Cohort over 2007 to 2024: A nutritionDay Descriptive Multivariable Analysis
Source: J Clin Med. 2026 Sep 14;15(18):7119. doi: 10.3390/jcm15187119 (PMC13607511; doi:10.3390/jcm15187119)
Supplement: Supplementary file 1 [file jcm-15-07119-s001.zip › PdfFile5sup_nday5_icu_en.pdf]

Patients list and outcomes

PLEASE KEEP LOCALLY ONLY!

A=still in hospital
B=Transferred to other hospital
C=transferred to longterm care
D=In rehabilitation center
F=discharged home
G=Dead
H=others

Center-Code | | | | | |

Unit-Code | | | | | |

		60 DAYS OUTCOME						
**Firstname, lastname, date of birth**	**Patient number**	**Outcome**	**Date of ICU discharge**	**Date of hospital discharge**	**Discharge diagnosis**	**Date of hospital readmission**	**Date of ICU readmission**	**Comments**
.. or patient sticker			(dd/mm/yyyy)	(dd/mm/yyyy)	ICD-10/ICD-9	(dd/mm/yyyy)	(dd/mm/yyyy)	
[redacted]	1	A	24.04.2017	02.05.2017	A41.9	12.05.2017	14.05.2017	

Thank you!

© nutritionDay - Authors: Hiesmayr / Schindler / Simon / Kiss / Kosak / Bauer - Medical University of Vienna / ESPEN Version 1 - 07.10.2016
