# Supplementary material for: Mortality, Renal Replacement Therapy and Renal Impairment Markers in Critically Ill Patients in a Multinational Point-Prevalence ICU Cohort over 2007 to 2024: A nutritionDay Descriptive Multivariable Analysis
Source: J Clin Med. 2026 Sep 14;15(18):7119. doi: 10.3390/jcm15187119 (PMC13607511; doi:10.3390/jcm15187119)
Supplement: Supplementary file 1 [file jcm-15-07119-s001.zip › WordFile_sup_inf_2.9.26.pdf]

Mortality, Renal Replacement Therapy and Renal Impairment Markers in Critically Ill Patients in a Multinational Point-Prevalence ICU Cohort over 2007 to 2024: A nutritionDay Descriptive Multivariable Analysis

Arabella Fischer-Hammerschmied [1,*], Peter Bauer [2], Fiona Trumshi [2], Silvia Tarantino [2,3], Emir Softic [2], Christian Schuh [4], Sylvia Ryz [1], Martin H. Bernardi [1], Andrea Lassnigg [1], Johanna Tichy [1], Sarah Yadavalli [1], Anna Schranz [1] and Michael Hiesmayr [2]

[1]Division of Cardiothoracic and Vascular Anaesthesia and Intensive Care Medicine, Department of Anaesthesia, Intensive Care and Pain Medicine, Medical University of Vienna, 1090 Vienna, Austria

[2]Center for Medical Data Science, Institute of Medical Statistics, Medical University of Vienna, 1090 Vienna, Austria

[3]Ludwig Boltzmann Institute for Arthritis and Rehabilitation, Medical University of Vienna, 1090 Vienna, Austria

[4]IT Systems and Communications, Medical University of Vienna, 1090 Vienna, Austria

*Corresponding Author: arabella.fischer-hammerschmied@meduniwien.ac.at

Tel: +43 1 40400 – 52620

Fax: +43 1 40400 – 41100

Währinger Gürtel 18-20

1090 Wien

Vienna General Hospital

Medical University of Vienna

Table of contents

Table S1. Baseline characteristics, renal impairment markers and mortality across different world regions

Table S2. Baseline characteristics and independent variables in patients with and without available mortality data

Table S3. Association between sixty-day hospital mortality and ICU- and patient-level factors in a multivariable logistic regression

Figure S1. Sensitivity analysis for sixty-day hospital mortality accounting for the bias of ICU length of stay

Figure S2. Sensitivity analysis for sixty-day hospital mortality with complete cases excluding patients with missing values for independent covariables or with imputation of both missing values for independent covariables and mortality

Figure S3. Sensitivity analysis for sixty-day hospital mortality with inverse probability weighting for mortality availability or with imputation of both missing values for independent covariables and mortality.

Figure S4. Sensitivity analysis including the original SAPS 2 score for sixty-day hospital mortality with complete cases excluding patients with missing values for independent covariables or with imputation of both missing values for independent covariables and mortality

Figure S5. Association between creatinine and sixty-day hospital mortality when using restricted cubic splines for creatinine, urine volume and adjusted SAPS 2 score

Figure S6. Association between urine volume and sixty-day hospital mortality when using restricted cubic splines for creatinine, urine volume and adjusted SAPS 2 score

Figure S7. Association between adjusted SAPS 2 score and sixty-day hospital mortality when using restricted cubic splines for creatinine, urine volume and adjusted SAPS 2 score

Table S4. Associations between renal replacement therapy and ICU- and patient-level factors in a multivariable logistic regression

Figure S8. Sensitivity analysis for renal replacement therapy with complete cases excluding patients with missing values for independent covariables or with imputation of missing values for independent covariables

Table S5. Mean deviations of serum creatinine at ICU admission from baseline creatinine for ICU- and patient-level factors in a multivariable linear regression

Figure S9. Sensitivity analysis for mean deviations of serum creatinine at ICU admission from baseline creatinine with complete cases excluding patients with missing values for independent covariables or with imputation of both missing values for independent covariables and creatinine

Table S6. Mean deviations of urine volume at ICU admission from baseline urine volume for ICU- and patient-level factors in a multivariable linear regression

Figure S10. Sensitivity analysis for mean deviations of urine volume at ICU admission from baseline urine volume with complete cases excluding patients with missing values for independent covariables or with imputation of both missing values for independent covariables and urine volume

Figure S11. Sensitivity analysis for higher versus lower urine volume (≥500 vs <500 ml) at ICU admission in a multivariable mixed-effects logistic regression

	Europe	North America	Latin America	Asia
N	11837 (50%)	1477 (6%)	4264 (18%)	5907 (25%)
**ICU-LEVEL FACTORS**				
Time Period				
2007-2011	4197 (35%)	421 (29%)	530 (12%)	1076 (18%)
2012-2016	3760 (32%)	959 (65%)	811 (19%)	2127 (36%)
2017-2021	1876 (16%)	97 (7%)	1250 (29%)	1248 (21%)
2022-2024	2004 (17%)	0 (0%)	1673 (39%)	1456 (25%)
Time from ICU admission to nutritionDay termed "ICU days" (days), mean (SD) median (Q1-Q3)	11 (SD 21) 4 (1-12)	7 (SD 13) 2 (1-6)	14 (SD 23) 6 (2-17)	11 (SD 21) 4 (1-12)
**PATIENT-LEVEL FACTORS AT ICU ADMISSION**				
Female (n)	4587 (39%)	647 (44%)	1883 (44%)	2342 (40%)
Age (years)	62.9 (SD 16.0)	60.8 (SD 17.0)	60.0 (SD 18.6)	59.6 (SD 17.5)
BMI (kg/m^2)	27.1 (SD 6.3)	29.0 (SD 8.3)	26.4 (SD 6.3)	25.2 (SD 5.3)
Creatinine at ICU admission (mg/dl) mean (SD) median (Q1-Q3)	1.44 (SD 1.31) 1.02 (0.74-1.60)	1.73 (SD 1.73) 1.16 (0.80-1.90)	1.45 (SD 1.39) 0.98 (0.70-1.52)	1.46 (SD 1.39) 1.00 (0.74-1.54)
BUN at ICU admission (mg/dl) mean (SD) median (Q1-Q3)	36.6 (SD 34.1) 25.0 (14.0-46.0)	30.1 (SD 25.2) 21.0 (14.0-38.0)	34.9 (SD 34.5) 22.0 (15.0-40.0)	32.7 (SD 28.8) 22.0 (14.0-42.0)
Urine volume in 24 hours at ICU admission (ml) mean (SD) median (Q1-Q3)	1766 (SD 1231) 1550 (900-2370)	1461 (SD 1212) 1150 (610-1975)	1375 (SD 1078) 1155 (600-1900)	1590 (SD 1091) 1400 (850-2083)
SAPS 2 score at ICU admission (points)	41.8 (SD 17.6)	35.4 (SD 15.7)	39.5 (SD 17.9)	37.0 (SD 17.5)
Adjusted SAPS 2 score at ICU admission without age and urine volume (points) mean (SD) median (Q1-Q3)	27.4 (SD 15.4) 25 (16-39)	21.6 (SD 13.9) 19 (11-30)	25.6 (SD 15.5) 23 (13-36)	23.8 (SD 15.0) 22 (12-33)
Reason for ICU dependency at ICU admission (n)				
Abdominal	2400 (20%)	185 (13%)	600 (14%)	693 (12%)
Burns	106 (0.9%)	1 (0.07%)	37 (0.9%)	30 (0.5%)
Cardiac	2751 (23%)	362 (25%)	759 (18%)	1327 (22%)
Neurologic	1959 (17%)	245 (17%)	921 (22%)	1245 (21%)

Pulmonary	3113 (26%)	572 (39%)	1106 (26%)	1485 (25%)
Sepsis	1979 (17%)	240 (16%)	683 (16%)	681 (12%)
Trauma	1008 (9%)	82 (6%)	311 (7%)	526 (9%)
Other	1526 (13%)	292 (20%)	542 (13%)	1114 (19%)
**VARIABLES ON NUTRITIONDAY**				
Renal Replacement Therapy on nutritionDay (n)	1124 (9.5%)	147 (10%)	464 (11%)	578 (10%)
SOFA score at nutritionDay (points)	4.3 (SD 3.3)	3.8 (SD 3.4)	2.4 (SD 2.7)	3.8 (SD 3.5)
Creatinine at nutritionDay (mg/dl)<br>median (Q1-Q3)	1.27 (SD 1.13)<br>0.90 (0.63-1.41)	1.59 (SD 1.54)<br>1.00 (0.71-1.76)	1.42 (SD 1.31)<br>0.98 (0.66-1.60)	1.41 (SD 1.31)<br>1.00 (0.68-1.60)
Urine volume in 24 hours at nutritionDay (ml)<br>mean (SD)<br>median (Q1-Q3)	2146 (SD 1383)<br>2000 (1200-2900)	1708 (SD 1339)<br>1456 (720-2420)	1718 (SD 1269)<br>1550 (800-2400)	1831 (SD 1185)<br>1650 (1062-2440)
No diuretics on nutritionDay (n)	7124 (60%)	1122 (76%)	3210 (75%)	4187 (71%)
Intermittent diuretics on nutritionDay (n)	1900 (16%)	63 (4%)	613 (14%)	790 (13%)
Continuous diuretics on nutritionDay (n)	2813 (24%)	292 (20%)	441 (10%)	930 (16%)
**VARIABLES AFTER NUTRITIONDAY**				
Time from ICU admission to hospital discharge (days)<br>mean (SD)<br>median (Q1-Q3)	30 (SD 28)<br>21 (10-41)	19 (SD 26)<br>12 (6-23)	29 (SD 28)<br>21 (10-40)	26 (SD 32)<br>15 (7-34)
60-day hospital mortality (n)	2294 (25%)	203 (16%)	793 (27%)	1119 (23%)

Table S1. Baseline characteristics, renal impairment markers and mortality across different world regions.

BUN: Blood Urea Nitrogen.

	Available information for Outcome Mortality	Missing information for Outcome Mortality	Total
N	18245 (77.7%)	5240 (22.3%)	23485 (100.0%)
**ICU-LEVEL FACTORS**			
**World Region**			
Europe (n)	9174 (50%)	2663 (51%)	11837 (50%)
North America (n)	1249 (7%)	228 (4%)	1477 (6%)
Latin America (n)	2974 (16%)	1290 (25%)	4264 (18%)
Asia (n)	4848 (27%)	1059 (20%)	5907 (25%)
**Time Period**			
2007-2011	4505 (25%)	1719 (33%)	6224 (27%)
2012-2016	5948 (33%)	1709 (33%)	7657 (33%)
2017-2021	3893 (21%)	578 (11%)	4471 (19%)
2022-2024	3899 (21%)	1234 (24%)	5133 (22%)
Time from ICU admission to nutritionDay termed “**ICU days**”, mean (SD) median (Q1-Q3)	11.0 (SD 20.7) 4 (1-12)	13.2 (SD 23.3) 5 (2-14)	11.5 (SD 21.3) 4 (1-13)
**ICU day groups**			
0-1	4243 (23%)	1130 (22%)	5373 (23%)
2-3	3491 (19%)	936 (18%)	4427 (19%)
4-6	2438 (13%)	640 (12%)	3078 (13%)
7-14	2922 (16%)	872 (17%)	3794 (16%)
≥15	3498 (19%)	1190 (23%)	4688 (20%)
missing	1653 (9%)	472 (9%)	2125 (9%)
**PATIENT-LEVEL FACTORS AT ICU ADMISSION**			
**Female** (n)	7256 (40%)	2203 (42%)	9459 (40%)
**Age** (years)	61.3 (SD 16.9)	61.7 (SD 17.5)	61.4 (SD 17.0)
Age groups			
[18-30)	1072 (6%)	343 (7%)	1415 (6%)
[30-40)	1233 (7%)	367 (7%)	1600 (7%)
[40-50)	1843 (10%)	483 (9%)	2326 (10%)
[50-60)	3103 (17%)	853 (16%)	3956 (17%)
[60-70)	4388 (24%)	1181 (23%)	5569 (24%)
[70-80)	4286 (23%)	1263 (24%)	5549 (24%)
[80-90)	2062 (11%)	653 (12%)	2715 (12%)
[90...)	258 (1%)	97 (2%)	355 (2%)
**BMI** (kg/m^2)	26.6 (SD 6.3)	26.6 (SD 6.3)	26.6 (SD 6.3)

	Available information for Outcome Mortality	Missing information for Outcome Mortality	Total
**BMI groups**			
[10-18.5)	841 (5%)	231 (4%)	1072 (5%)
[18.5-25)	7096 (39%)	1993 (38%)	9089 (39%)
[25-30)	5779 (32%)	1645 (31%)	7424 (32%)
[30-35)	2310 (13%)	653 (12%)	2963 (13%)
[35-40)	894 (5%)	228 (4%)	1122 (5%)
[40...)	618 (3%)	188 (4%)	806 (3%)
missing	707 (4%)	302 (6%)	1009 (4%)
**Creatinine at ICU admission** (mg/dl)<br>mean (SD)<br>median (Q1-Q3)	1.47 (SD 1.37)<br>1.01 (0.75-1.60)	1.46 (SD 1.39)<br>1.00 (0.72-1.58)	1.47 (SD 1.37)<br>1.00 (0.74-1.60)
**Creatinine at ICU admission groups**			
Q1:[0.1 - 0.7)	3156 (17%)	967 (18%)	4123 (18%)
Q2:[0.7 - 0.9)	3179 (17%)	922 (18%)	4101 (17%)
Q3:[0.9 - 1.2]	3410 (19%)	918 (18%)	4328 (18%)
Q4:[1.2 - 1.8]	3356 (18%)	904 (17%)	4260 (18%)
Q5:[1.8 - 15]	3309 (18%)	933 (18%)	4242 (18%)
missing	1835 (10%)	596 (11%)	2431 (10%)
BUN at ICU admission (mg/dl)<br>mean (SD)<br>median (Q1-Q3)	33.2 (SD 30.4)<br>23.0 (14.0-41.5)	35.2 (SD 32.5)<br>23.0 (14.0-44.0)	33.7 (SD 30.9)<br>23.0 (14.0-42.0)
**Urine volume in 24 hours at ICU admission** (ml), mean (SD)<br>median (Q1-Q3)	1633.66 (SD 1171.71)<br>1410.00 (800.00-2200.00)	1670.90 (SD 1216.05)<br>1480.00 (800.00-2250.00)	1641.87 (SD 1181.70)<br>1430.00 (800.00-2200.00)
**Urine volume at ICU admission groups**			
[0-670)	3204 (18%)	936 (18%)	4140 (18%)
[670-1200)	3313 (18%)	842 (16%)	4155 (18%)
[1200-1700)	3144 (17%)	898 (17%)	4042 (17%)
[1700-2460)	3368 (18%)	937 (18%)	4305 (18%)
[2460-9800)	3148 (17%)	963 (18%)	4111 (18%)
missing	2068 (11%)	664 (13%)	2732 (12%)
SAPS 2 score at ICU admission (points)	39.6 (SD 17.6)	40.6 (SD 17.9)	39.8 (SD 17.7)
**Adjusted SAPS 2 score at ICU admission without age and urine volume** (points)	25.6 (SD 15.4)	26.5 (SD 15.4)	25.8 (SD 15.4)
**Adjusted SAPS 2 score groups**			

	Available information for Outcome Mortality	Missing information for Outcome Mortality	Total
Q1	3617 (20%)	938 (18%)	4555 (19%)
Q2	3251 (18%)	894 (17%)	4145 (18%)
Q3	3795 (21%)	1099 (21%)	4894 (21%)
Q4	3927 (22%)	1192 (23%)	5119 (22%)
Q5	3655 (20%)	1117 (21%)	4772 (20%)
**Reason for ICU dependency at ICU admission** (n)			
Abdominal	3022 (17%)	856 (16%)	3878 (17%)
Burns	139 (0.8%)	35 (0.7%)	174 (0.7%)
Cardiac	4160 (23%)	1039 (20%)	5199 (22%)
Neurologic	3280 (18%)	1090 (21%)	4370 (19%)
Pulmonary	4759 (26%)	1517 (29%)	6276 (27%)
Sepsis	2743 (15%)	840 (16%)	3583 (15%)
Trauma	1533 (8%)	394 (8%)	1927 (8%)
Other	2731 (15%)	743 (14%)	3474 (15%)
**VARIABLES ON NUTRITIONDAY**			
**Renal Replacement Therapy on nutritionDay** (n)	1823 (10%)	490 (9%)	2313 (10%)
Hemofiltration	244 (1%)	75 (1%)	319 (1%)
Hemodialysis	1190 (7%)	312 (6%)	1502 (6%)
Hemodiafiltration	389 (2%)	103 (2%)	492 (2%)
SOFA score at nutritionDay (points)	3.8 (SD 3.3)	3.8 (SD 3.3)	3.8 (SD 3.3)
Creatinine at nutritionDay (mg/dl)<br>mean (SD)<br>median (Q1-Q3)	1.34 (SD 1.21)<br>0.94 (0.67-1.50)	1.36 (SD 1.30)<br>0.90 (0.62-1.52)	1.34 (SD 1.23)<br>0.93 (0.66-1.50)
Urine volume in 24 hours at nutritionDay (ml)<br>mean (SD)<br>median (Q1-Q3)	1961.00 (SD 1313.90)<br>1800.00 (1064.00-2670.00)	2000.69 (SD 1378.91)<br>1810.00 (1029.00-2715.50)	1969.64 (SD 1328.40)<br>1800.00 (1055.00-2690.00)
No diuretics on nutritionDay (n)	12165 (67%)	3478 (66%)	15643 (67%)
Intermittent diuretics on nutritionDay (n)	2625 (14%)	741 (14%)	3366 (14%)
Continuous diuretics on nutritionDay (n)	3455 (19%)	1021 (19%)	4476 (19%)
**VARIABLES AFTER NUTRITIONDAY**			
Time from ICU admission to hospital discharge (days), mean (SD)<br>median (Q1-Q3)	27.9 (SD 29.4)<br>18 (9-38)	24.2 (SD 19.7)<br>18 (9-34)	27.9 (SD 29.4)<br>18 (9-38)
**Outcome at 60 days after nutritionDay (n)**			

	Available information for Outcome Mortality	Missing information for Outcome Mortality	Total
**60-day hospital mortality**	4409 (24%)	N.A.	4409 (24%)
Still in hospital	1725 (9%)	N.A.	1725 (9%)
Transfer hospital	1347 (7%)	N.A.	1347 (7%)
Transfer long-term	773 (4%)	N.A.	773 (4%)
Rehabilitation	879 (5%)	N.A.	879 (5%)
Discharge home	8777 (48%)	N.A.	8777 (48%)
Other	335 (2%)	N.A.	335 (2%)

Table S2. Baseline characteristics and independent variables in patients with and without available mortality data.

N.A.: not available

		OR [95% CI] for mortality
Region	Europe	1.00 [1.00 1.00]
	North America	0.68** [0.52 0.91]
	Latin America	1.30** [1.08 1.57]
	Asia	1.12 [0.96 1.32]
Year	[2007-2011]	1.00 [1.00 1.00]
	[2012-2016]	0.80** [0.70 0.92]
	[2017-2021]	0.93 [0.79 1.10]
	[2022-2024]	0.77** [0.65 0.91]
ICU Days (days)	(0-1)	0.57*** [0.49 0.65]
	(2-3)	0.73*** [0.64 0.84]
	(4-6)	1.00 [1.00 1.00]
	(7-14)	1.08 [0.94 1.23]
	(15-..)	1.20** [1.05 1.37]
	missing	0.73*** [0.61 0.87]
Sex	female	1.00 [1.00 1.00]
	male	1.08 [0.99 1.17]
Age (years)	[18-30)	0.45*** [0.36 0.55]
	[30-40)	0.48*** [0.40 0.59]
	[40-50)	0.61*** [0.52 0.72]
	[50-60)	0.72*** [0.64 0.82]
	[60-70)	1.00 [1.00 1.00]
	[70-80)	1.36*** [1.22 1.52]
	[80-90)	1.87*** [1.64 2.14]
	[90...)	2.28*** [1.68 3.09]
BMI (kg/m^2)	[10-18.5)	1.31** [1.09 1.57]
	[18.5-25)	1.00 [1.00 1.00]
	[25-30)	0.83*** [0.76 0.91]
	[30-35)	0.81*** [0.71 0.92]
	[35-40)	0.87 [0.72 1.05]
	[40...)	0.97 [0.78 1.21]
	missing	1.09 [0.88 1.37]
RRT	RRT no	1.00 [1.00 1.00]
	RRT yes	2.07*** [1.82 2.34]
Creatinine ICU Day 0	Q1:[0.1 - 0.7)	0.99 [0.86 1.13]
(mg/dl)	Q2:[0.7 - 0.9)	1.07 [0.93 1.23]
	Q3:[0.9 - 1.2]	1.00 [1.00 1.00]
	Q4:[1.2 - 1.8]	1.34*** [1.18 1.52]
	Q5:[1.8 - 15]	1.35*** [1.19 1.54]
	missing	1.04 [0.88 1.24]
Urine Volume ICU Day 0	[0-670)	1.18* [1.04 1.35]
(ml)	[670-1200)	1.03 [0.90 1.17]
	[1200-1700)	1.00 [1.00 1.00]
	[1700-2460)	0.87* [0.76 0.99]
	[2460-9800)	0.85* [0.74 0.97]
	missing	1.18 [1.00 1.39]
SAPS 2 adjusted	Q1	0.43*** [0.37 0.50]

	Q2	0.70*** [0.61 0.80]
	Q3	1.00 [1.00 1.00]
	Q4	1.46*** [1.30 1.63]
	Q5	2.21*** [1.97 2.48]
ICU dependency	no	1.00 [1.00 1.00]
	abdominal	1.04 [0.92 1.17]
	no	1.00 [1.00 1.00]
	burns	1.20 [0.73 1.96]
	no	1.00 [1.00 1.00]
	cardiac	0.85** [0.76 0.96]
	no	1.00 [1.00 1.00]
	neurologic	1.08 [0.96 1.22]
	no	1.00 [1.00 1.00]
	pulmonary	1.40*** [1.26 1.54]
	no	1.00 [1.00 1.00]
	septic	1.36*** [1.22 1.51]
	no	1.00 [1.00 1.00]
	trauma	0.65*** [0.54 0.77]
	no	1.00 [1.00 1.00]
	other	0.97 [0.86 1.11]
Constant		0.24*** [0.19 0.31]
Random intercept variance		1.54*** [1.41 1.68]
Observations		18245

Table S3. Association between sixty-day hospital mortality and ICU- and patient-level factors in a multivariable logistic regression.

* p < 0.05, ** p < 0.01, *** p < 0.001

Odds ratios (OR) relative to the reference category (OR=1) are presented with 95% confidence intervals.

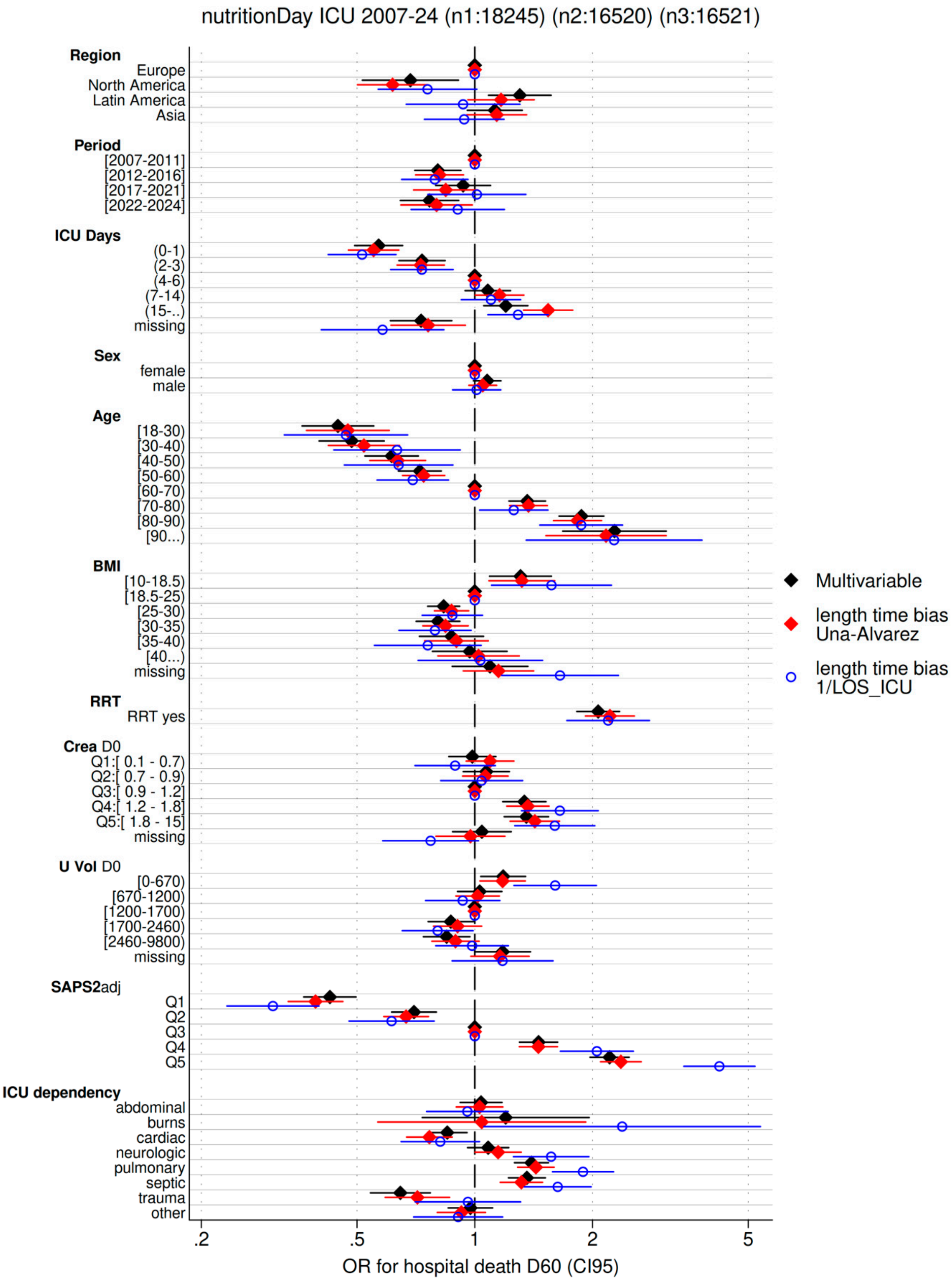

Figure S1. Sensitivity analysis for sixty-day hospital mortality accounting for the bias of ICU length of stay with length-of-stay weighting algorithm for cross-sectional studies described by De Uña-

Álvarez* (n2=16520 in red) or patients weighted by 1/ICU length of stay, when ICU length of stay was available (n3=16521 in blue).

Hospital death D60: sixty-day hospital mortality; ICU Days in days; Age in years; BMI in kg/m^2; RRT: Renal Replacement Therapy; Crea D0: Creatinine at ICU admission in mg/dl; U Vol D0: Urine volume at ICU admission in ml; SAPS 2adj: Adjusted SAPS 2 score without age and urine-volume components

*Frantal S, Pernicka E, Hiesmayr M, Schindler K, Bauer P. Length bias correction in one-day cross-sectional assessments - The nutritionDay study. Clin Nutr. 2016;35(2):522-7. https://doi.org/10.1016/j.clnu.2015.03.019

*De Uña-álvarez J. Nonparametric estimation under length-biased sampling and Type I censoring: A moment based approach. Annals of the Institute of Statistical Mathematics. 2004;56(4):667-81. https://doi.org/10.1007/BF02506482

R and Stata Code for sensitivity analysis for sixty-day hospital mortality accounting for the bias of ICU length of stay with length-of-stay weighting algorithm for cross-sectional studies described by De Uña-Álvarez* or with patients weighted by ICU length of stay:

R Code for with length-of-stay weighting algorithm described by De Uña-Álvarez*:

```
“data$zens<-(data$outcome_code_neu==1)*1
data$w_i<-1/nrow(data)
data$w_i[which(data$hospital_stay_y>30)]<-
data$hospital_stay_y[which(data$hospital_stay_y>30)]/ (nrow(data)*30)
data$w_i[data$zens==1]<-0
data$w_i_schlange<-data$w_i*(1/data$hospital_stay_y)
data$w_i_schlange<-data$w_i_schlange/sum(data$w_i_schlange,na.rm = T)
data$zens<-NULL”
```

Stata Continuation Code for length-of-stay weighting algorithm described by De Uña-Álvarez*::

```
“local var deceased

meglm `var'                                                    ///
            ib(#1).region_un                                   /// *Region
            ib(#1).year_period                                 /// *Period
            ib(#3).icutime_g5bis                            /// *ICU days (=time from
ICU admission to nutritionDay) in 5 groups
            ib(#1).sex                                         /// *Sex
            ib(#5).age_group10                                 /// *Age groups, each age
group including a life span of about 10 years
            ib(#2).bmi_who2_center                                  /// *BMI WHO
categories
            ib(#1).replace_simple                           /// *RRT yes
            ib(#3).creatinine_day_max_u1_g5_bis   /// *Creatinine quintiles
            ib(#3).urine_output_max_n_g5_bis                /// *Urine volume quintiles
            ib(#3).saps2_new_no_age_no_urine_g5             /// *Adjusted SAPS 2
quintiles
            ib(#1).icu_dependency_abdominal                 /// *Reasons for ICU
dependency at ICU admission
            ib(#1).icu_dependency_burns                     ///
            ib(#1).icu_dependency_cardiac                   ///
            ib(#1).icu_dependency_neurological              ///
            ib(#1).icu_dependency_pulmonary                 ///
            ib(#1).icu_dependency_septic                    ///
            ib(#1).icu_dependency_trauma                    ///
            ib(#1).icu_dependency_others                    ///
            [pw=w_i_schlange]                                  /// *Weighting algorithm
described by De Uña-Álvarez, see R code above*
            || unit:,                                              /// *Random factor:
Individual ICU
            family(binomial)                                   ///
            link(logit)

est store e_1_random_pwi
est save e_1_random_pwi, replace
```

Stata Code for pragmatic length-of-stay weighting by ICU length of stay:2131

```
Gen losicu_weight=1/los_icu

local var deceased
meglm `var'                                                    ///
            ib(#1).region_un                                   ///
            ib(#1).year_period                                 ///
            ib(#3).icutime_g5bis                            ///
            ib(#1).sex                                         ///
            ib(#5).age_group10                                 ///
```

```
ib(#2).bmi_who2_center ///
ib(#1).replace_simple ///
ib(#3).creatinine_day_max_u1_g5_bis ///
ib(#3).urine_output_max_n_g5_bis ///
ib(#3).saps2_new_no_age_no_urine_g5 ///
ib(#1).icu_dependency_abdominal ///
ib(#1).icu_dependency_burns ///
ib(#1).icu_dependency_cardiac ///
ib(#1).icu_dependency_neurological ///
ib(#1).icu_dependency_pulmonary ///
ib(#1).icu_dependency_septic ///
ib(#1).icu_dependency_trauma ///
ib(#1).icu_dependency_others ///
[pw=losicu_weight] /// *Patients' Weighting
by ICU length of stay
|| unit:, /// *Random factor:
Individual ICU
family(binomial) ///
link(logit)

est store e_1_random_pw_los
est save e_1_random_pw_los, replace"
```

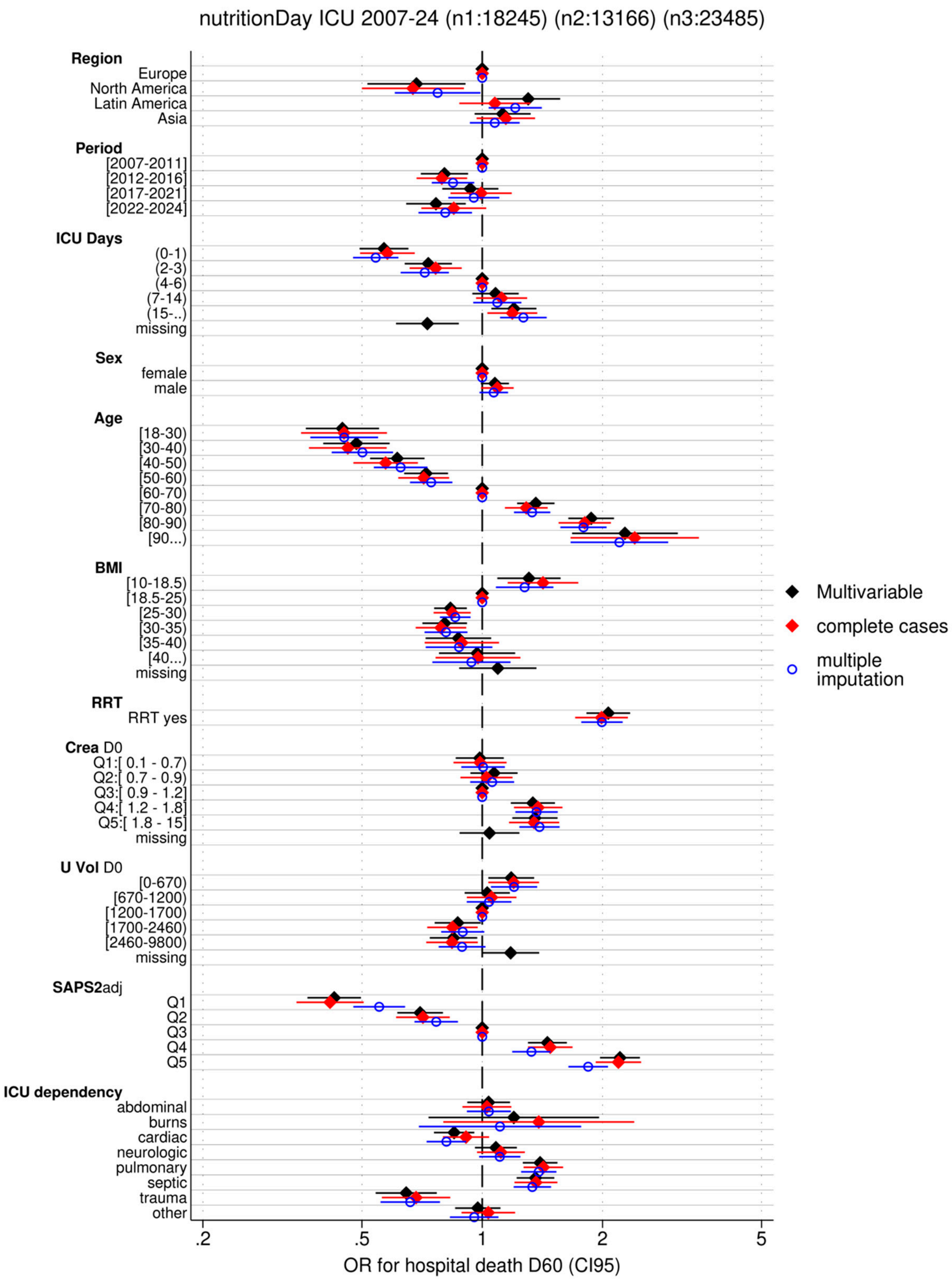

Figure S2. Sensitivity analysis for sixty-day hospital mortality with complete cases excluding patients with missing values for independent covariables (n2=13166 in red) or with imputation of both

missing values for independent covariables and mortality (n3=23485 in blue).

Missing values for independent covariables included missing ICU days, BMI, creatinine at ICU admission, urine volume at ICU admission, or more than three missing SAPS 2 components.

Hospital death D60: sixty-day hospital mortality; ICU Days in days; Age in years; BMI in kg/m^2; RRT: Renal Replacement Therapy; Crea D0: Creatinine at ICU admission in mg/dl; U Vol D0: Urine volume at ICU admission in ml; SAPS 2adj: Adjusted SAPS 2 score without age and urine-volume components

Stata code for sensitivity analysis for sixty-day hospital mortality with complete cases excluding patients with missing values for independent covariables or with imputation of both missing values for independent covariables and mortality:

```
"***** meglm for variable deceased (primary analysis for mortality)

local var deceased
meglm `var'                                                    ///
                ib(#1).region_un                               /// *Region
                ib(#1).year_period                        /// *Period
                ib(#3).icutime_g5bis                      /// *ICU days (=time from
ICU admission to nutritionDay) in 5 groups
                ib(#1).sex                                     /// *Sex
                ib(#5).age_group10                        /// *Age groups, each age
group including a life span of about 10 years
                ib(#2).bmi_who2_center                         /// *BMI WHO
categories
                ib(#1).replace_simple                     /// *RRT yes
                ib(#3).creatinine_day_max_u1_g5_bis       /// *Creatinine quintiles
                ib(#3).urine_output_max_n_g5_bis          /// *Urine volume quintiles
                ib(#3).saps2_new_no_age_no_urine_g5            /// *Adjusted SAPS 2
quintiles
                ib(#1).icu_dependency_abdominal           /// *Reasons for ICU
dependency at ICU admission
                ib(#1).icu_dependency_burns               ///
                ib(#1).icu_dependency_cardiac             ///
                ib(#1).icu_dependency_neurological        ///
                ib(#1).icu_dependency_pulmonary           ///
                ib(#1).icu_dependency_septic              ///
                ib(#1).icu_dependency_trauma              ///
                ib(#1).icu_dependency_others              ///
                || unit:,                                      /// *Random factor:
Individual ICU
                family(binomial)                               ///
```

```
                link(logit)


***** sensitivity analysis: complete cases for mortality
drop if var_miss==1

local var deceased
meglm `var'                                                     ///
                ib(#1).region_un                                ///
                ib(#1).year_period                         ///
                ib(#3).icutime_g5bis                       ///
                ib(#1).sex                                      ///
                ib(#5).age_group10                         ///
                ib(#2).bmi_who2_center                          ///
                ib(#1).replace_simple                      ///
                ib(#3).creatinine_day_max_u1_g5_bis        ///
                ib(#3).urine_output_max_n_g5_bis           ///
                ib(#3).saps2_new_no_age_no_urine_g5             ///
                ib(#1).icu_dependency_abdominal            ///
                ib(#1).icu_dependency_burns                ///
                ib(#1).icu_dependency_cardiac              ///
                ib(#1).icu_dependency_neurological         ///
                ib(#1).icu_dependency_pulmonary            ///
                ib(#1).icu_dependency_septic               ///
                ib(#1).icu_dependency_trauma               ///
                ib(#1).icu_dependency_others               ///
                || unit:,                                       ///
                family(binomial)                                ///
                link(logit)


******* sensitivity analysis: multiple imputation for mortality
******* version 2 2025_08_14

mi set mlong

*******
mi register imputed deceased icutime_g5bis bmi_who2_center creatinine_day_max_u1_g5_bis
urine_output_max_n_g5_bis saps2_new_no_age_no_urine_g5 icu_dependency_abdominal
icu_dependency_burns icu_dependency_cardiac icu_dependency_neurological
icu_dependency_pulmonary icu_dependency_septic icu_dependency_trauma
icu_dependency_others age_group10 replace_simple region_un year_period sex

mi impute chained                               ///
        (logit) deceased                                ///
        (mlogit) icutime_g5bis bmi_who2_center creatinine_day_max_u1_g5_bis
urine_output_max_n_g5_bis  saps2_new_no_age_no_urine_g5         ///
        = region_un year_period sex age_group10 replace_simple icu_dependency_abdominal
icu_dependency_burns icu_dependency_cardiac icu_dependency_neurological
icu_dependency_pulmonary icu_dependency_septic icu_dependency_trauma
icu_dependency_others ,        ///
```

```
add(25) rseed(25178) savetrace(trace1, replace)


****** meglm with imputed datafile
clear
use `datadir'`datafile_i'.dta

local var deceased
mi estimate, cmdok: meglm `var'                                   ///
                  ib(#1).region_un                                      ///
                  ib(#1).year_period                              ///
                  ib(#3).icutime_g5bis                            ///
                  ib(#1).sex                                            ///
                  ib(#5).age_group10                              ///
                  ib(#2).bmi_who2_center                                ///
                  ib(#1).replace_simple                           ///
                  ib(#3).creatinine_day_max_u1_g5_bis             ///
                  ib(#3).urine_output_max_n_g5_bis                ///
                  ib(#3).saps2_new_no_age_no_urine_g5                   ///
                  ib(#1).icu_dependency_abdominal                 ///
                  ib(#1).icu_dependency_burns                     ///
                  ib(#1).icu_dependency_cardiac                   ///
                  ib(#1).icu_dependency_neurological              ///
                  ib(#1).icu_dependency_pulmonary                 ///
                  ib(#1).icu_dependency_septic                    ///
                  ib(#1).icu_dependency_trauma                    ///
                  ib(#1).icu_dependency_others                    ///
                  || unit:,                                             ///
                  family(binomial)                                      ///
                  link(logit)                                      ”
```

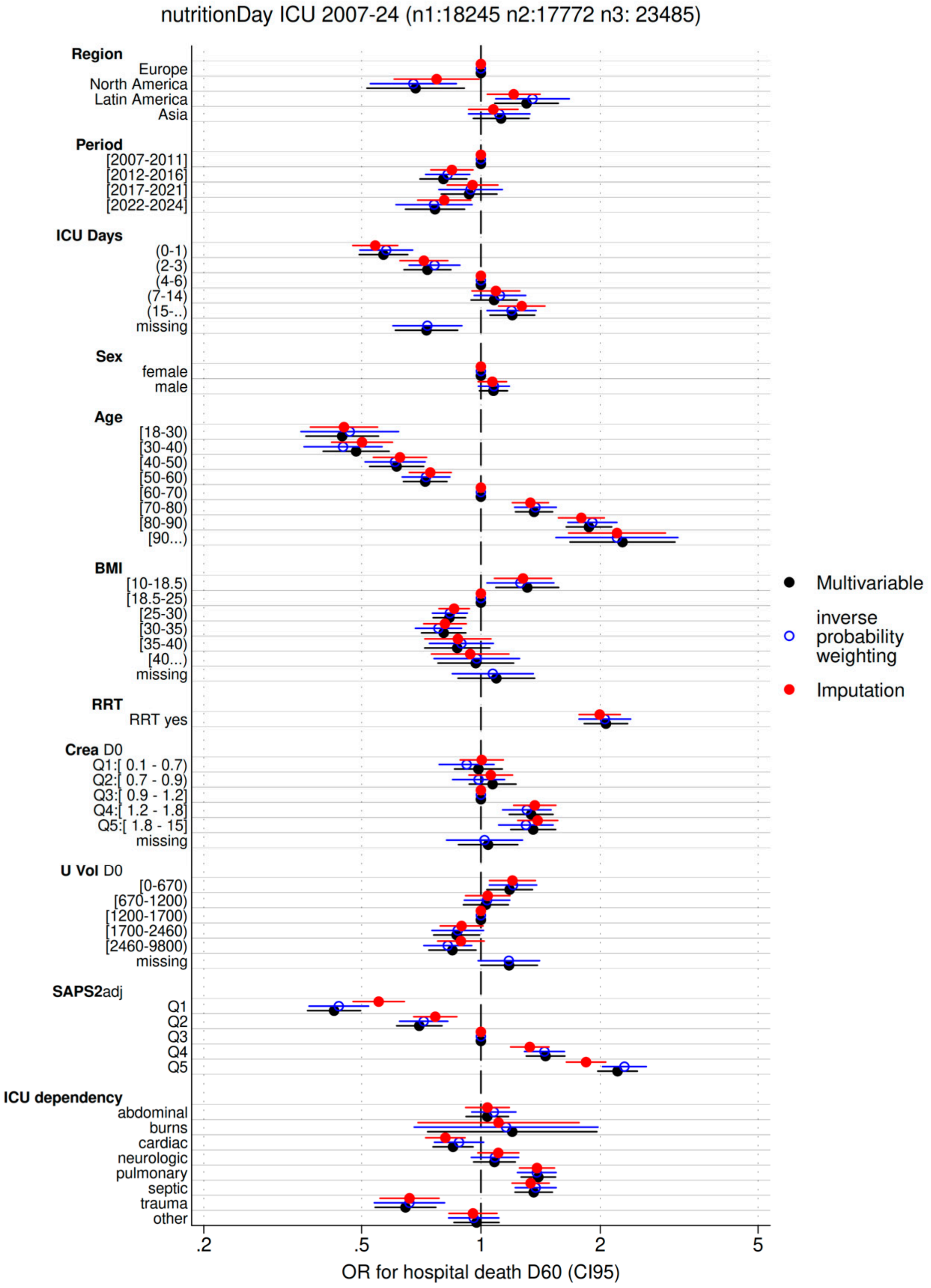

Figure S3. Sensitivity analysis for sixty-day hospital mortality with inverse probability weighting for mortality availability (n2=17772 in blue) or with imputation of both missing values for independent covariables and mortality (n3=23485 in red).

Hospital death D60: sixty-day hospital mortality; ICU Days in days; Age in years; BMI in kg/m^2; RRT: Renal Replacement Therapy; Crea D0: Creatinine at ICU admission in mg/dl; U Vol D0: Urine volume at ICU admission in ml; SAPS 2adj: Adjusted SAPS 2 score without age and urine-volume components

Stata Code for sensitivity analysis for sixty-day hospital mortality with inverse probability weighting for mortality availability:

```
"***** determine ipw and rerun analysis

**** variable 0 when outcome is missing, 1 when outcome deceased not missing
gen byte observed=!missing(deceased)
tab deceased observed, missing

**** recruted patients
sort year_unit
by year_unit: gen patient_total=_N
by year_unit: gen count_year_unit=_n
egen patient_total_g5=cut(patient_total), group(5)

encode country_code, gen(country_numeric)
**********
glm observed                                            ///
        ib(#1).year_z                                   /// *each year from 2007-
2024
        ib(#7).country_numeric                          /// *each country
        ib(#3).icu_type                                 /// *ICU types: medical ICU;
surgical ICU; interdisciplinary ICU;  high dependency unit; other ICU type
        ib(#3).patient_total_g5                   /// *number of recruited patients per
ICU in 5 groups
        ib(#1).saps2_nmiss                              /// *number of missing
components in original SAPS 2 score
        ib(#3).icutime_g5bis                            /// *ICU days (=time from
ICU admission to nutritionDay) in 5 groups
        ib(#1).sex                                              ///
        ib(#5).age_group10                              /// *Age groups, each age
group including a life span of about 10 years
        ib(#1).icu_dependency_abdominal                 /// *Reasons for ICU
dependency at ICU admission
        ib(#1).icu_dependency_burns                     ///
        ib(#1).icu_dependency_cardiac                   ///
        ib(#1).icu_dependency_neurological              ///
        ib(#1).icu_dependency_pulmonary                 ///
        ib(#1).icu_dependency_septic                    ///
        ib(#1).icu_dependency_trauma                    ///
        ib(#1).icu_dependency_others                    ///
        ,                                                       ///
```

```
                    family(binomial)                                    ///
                    link(logit)
est store miss_model2
est save miss_model2, replace
predict p_observed_miss_model2, mu
gen ipw_miss_model2=1/p_observed_miss_model2 if observed==1

****************** model2


local var deceased
meglm `var'                                                             ///
                    ib(#1).region_un                                    /// *Region
                    ib(#1).year_period                          /// *Period
                    ib(#3).icutime_g5bis                        /// *ICU days (=time from
ICU admission to nutritionDay) in 5 groups
                    ib(#1).sex                                          /// *Sex
                    ib(#5).age_group10                          /// *Age groups, each age
group including a life span of about 10 years
                    ib(#2).bmi_who2_center                              /// *BMI WHO
categories
                    ib(#1).replace_simple                       /// *RRT yes
                    ib(#3).creatinine_day_max_u1_g5_bis         /// *Creatinine quintiles
                    ib(#3).urine_output_max_n_g5_bis            /// *Urine volume quintiles
                    ib(#3).saps2_new_no_age_no_urine_g5                 /// *Adjusted SAPS 2
quintiles
                    ib(#1).icu_dependency_abdominal             /// *Reasons for ICU
dependency at ICU admission
                    ib(#1).icu_dependency_burns                 ///
                    ib(#1).icu_dependency_cardiac               ///
                    ib(#1).icu_dependency_neurological          ///
                    ib(#1).icu_dependency_pulmonary             ///
                    ib(#1).icu_dependency_septic                ///
                    ib(#1).icu_dependency_trauma                ///
                    ib(#1).icu_dependency_others                ///
                    [pweight=ipw_miss_model2]                   ///
                    || unit:                                            /// *Random factor:
Individual ICU
                    ,                                                   ///
                    family(binomial)                                    ///
                    link(logit)

est store e_1_random_ipw2
est save e_1_random_ipw2, replace”
```

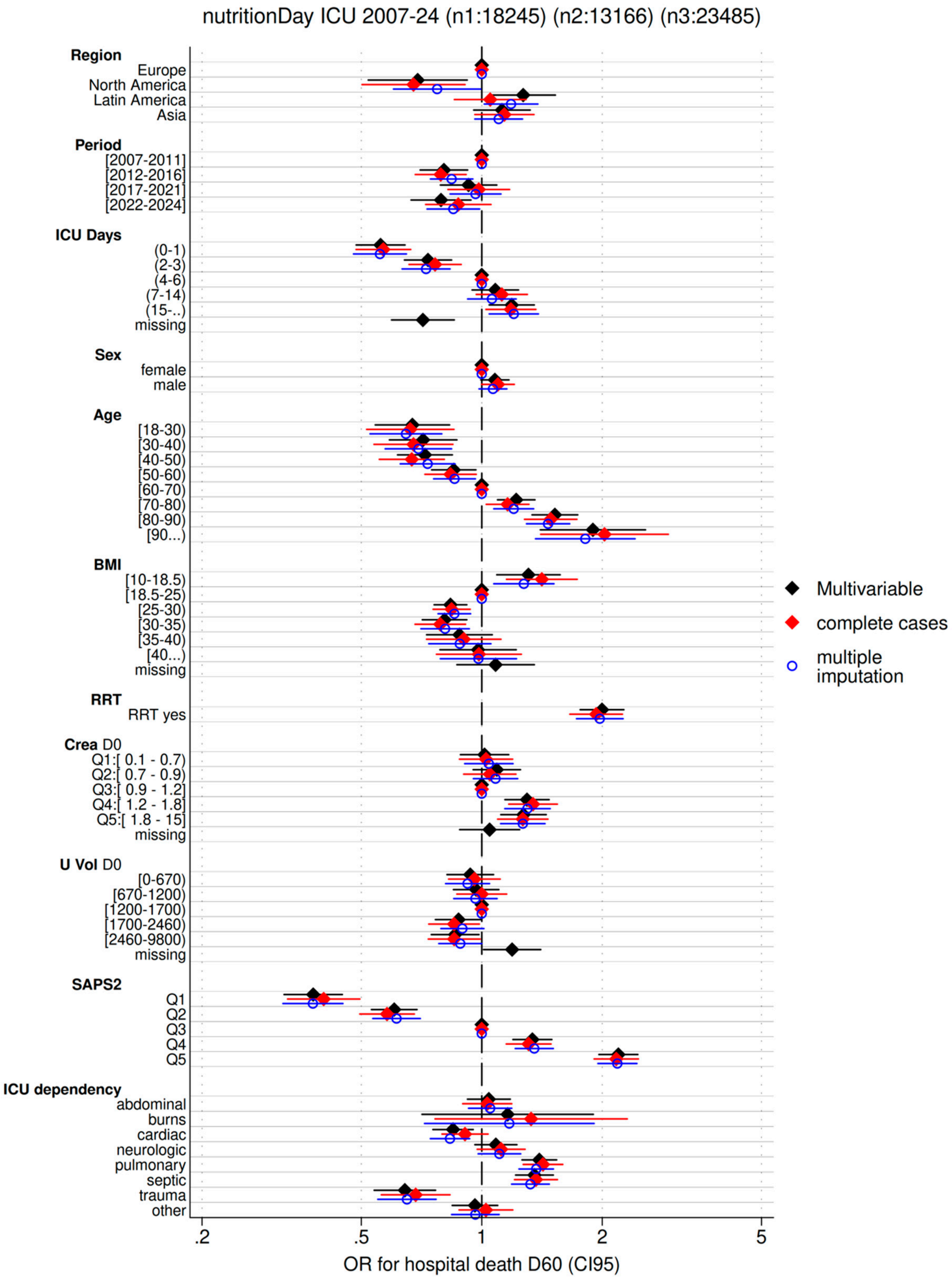

Figure S4. Sensitivity analysis including the original SAPS 2 score for sixty-day hospital mortality with complete cases excluding patients with missing values for independent covariables (n2=13166 in

red) or with imputation of both missing values for independent covariables and mortality (n3=23485 in blue). Here, the original SAPS 2 score, rather than the adjusted SAPS 2 score, was included in the multivariable model. This may introduce collinearity and mathematical coupling, because both age and urine volume are components of the original SAPS 2 score and were also included as separate independent variables in the model.

Missing values for independent covariables included missing ICU days, BMI, creatinine at ICU admission, urine volume at ICU admission, or any missing SAPS 2 component.

Hospital death D60: sixty-day hospital mortality; ICU Days in days; Age in years; BMI in kg/m^2; RRT: Renal Replacement Therapy; Crea D0: Creatinine at ICU admission in mg/dl; U Vol D0: Urine volume at ICU admission in ml; SAPS 2: Original SAPS 2 score

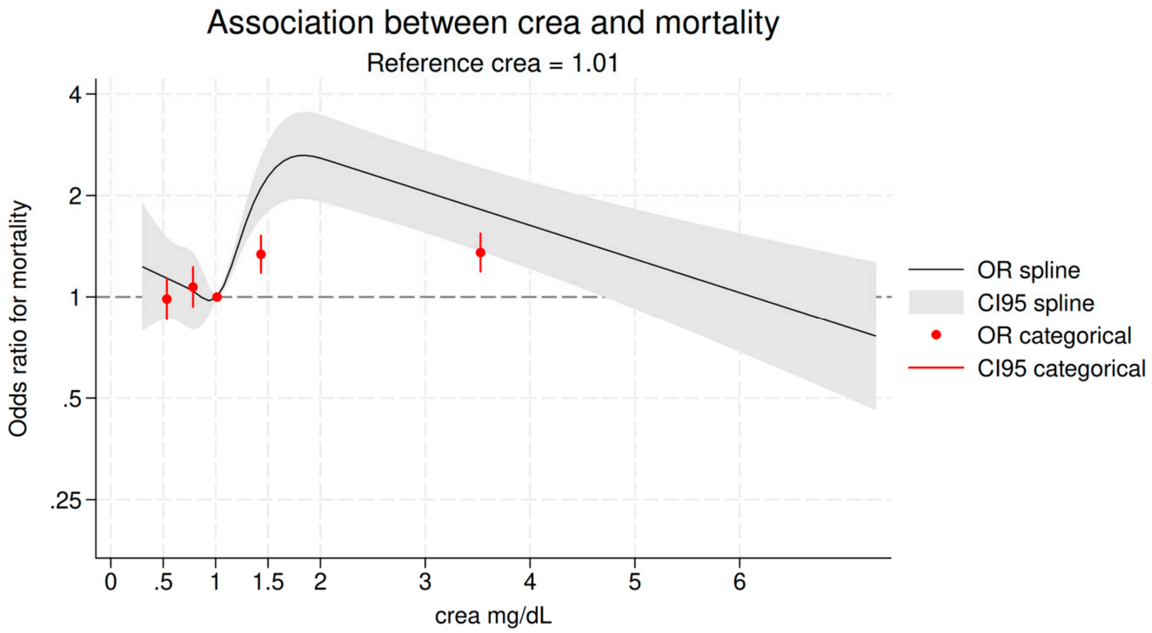

Figure S5. Association between creatinine and sixty-day hospital mortality when using restricted cubic splines for creatinine, urine volume and adjusted SAPS 2 score (n=14802)

This sensitivity multivariable analysis illustrates the association between creatinine and mortality while accounting for all other covariables (Region, Period, ICU Days, Sex, Age, BMI, Urine Volume, adjusted SAPS 2, Reasons for ICU dependency) with the individual ICU as random effect. Reference

creatinine, expressed as the mean when all covariables are in their reference groups, was 1.01 mg/dl in the multivariable model (n=14802). OR from treating creatinine continuously using restricted cubic splines (black line) is compared with OR from categorising creatinine (red dots).

Stata code for sensitivity analysis for sixty-day hospital mortality using restricted cubic splines for creatinine and adjusted SAPS 2 score:

```
“***** mortality with adjusted saps2 values

makespline rcs saps2_new_no_age_no_urine, knots(5) basis(saps2_s)
matrix K_saps2 = r(knots)
matrix R_saps2 = r(minmax)
rename _rs_rcs_1 saps2_s_0

makespline rcs creatinine_day_max_u1, knots(5) basis(crea_s)
matrix K_crea = r(knots)
matrix R_crea = r(minmax)
rename _rs_rcs_1 crea_s_0

makespline rcs urine_output_max_n, knots(5) basis(uvol_s)
matrix K_uvol = r(knots)
matrix R_uvol = r(minmax)
rename _rs_rcs_1 uvol_s_0

meglm deceased ///
   ib(#1).region_un /// *Region
   ib(#1).year_period /// *Period
   ib(#3).icutime_g5bis /// *ICU days (=time from ICU admission to nutritionDay) in 5 groups
   ib(#1).sex /// *Sex
   ib(#5).age_group10 /// *Age groups, each age group including a life span of about 10 years
   ib(#2).bmi_who2_center /// *BMI WHO categories
   ib(#1).replace_simple /// *RRT yes
   c.saps2_s_0 c.saps2_s_1_1 c.saps2_s_1_2 c.saps2_s_1_3 /// *Adjusted SAPS 2 score modeled as
continuous variable using restricted cubic splines
   c.crea_s_0 c.crea_s_1_1 c.crea_s_1_2 c.crea_s_1_3 /// *Creatinine modeled as continuous
variable using restricted cubic splines
   c.uvol_s_0 c.uvol_s_1_1 c.uvol_s_1_2 c.uvol_s_1_3 ///*Urine Volume modeled as continuous
variable using restricted cubic splines
   ib(#1).icu_dependency_abdominal ///  *Reasons for ICU dependency at ICU admission
   ib(#1).icu_dependency_burns ///
   ib(#1).icu_dependency_cardiac ///
   ib(#1).icu_dependency_neurological ///
   ib(#1).icu_dependency_pulmonary ///
   ib(#1).icu_dependency_septic ///
   ib(#1).icu_dependency_trauma ///
   ib(#1).icu_dependency_others ///
```

|| unit: /// *Random factor: Individual ICU
, family(binomial) link(logit)”

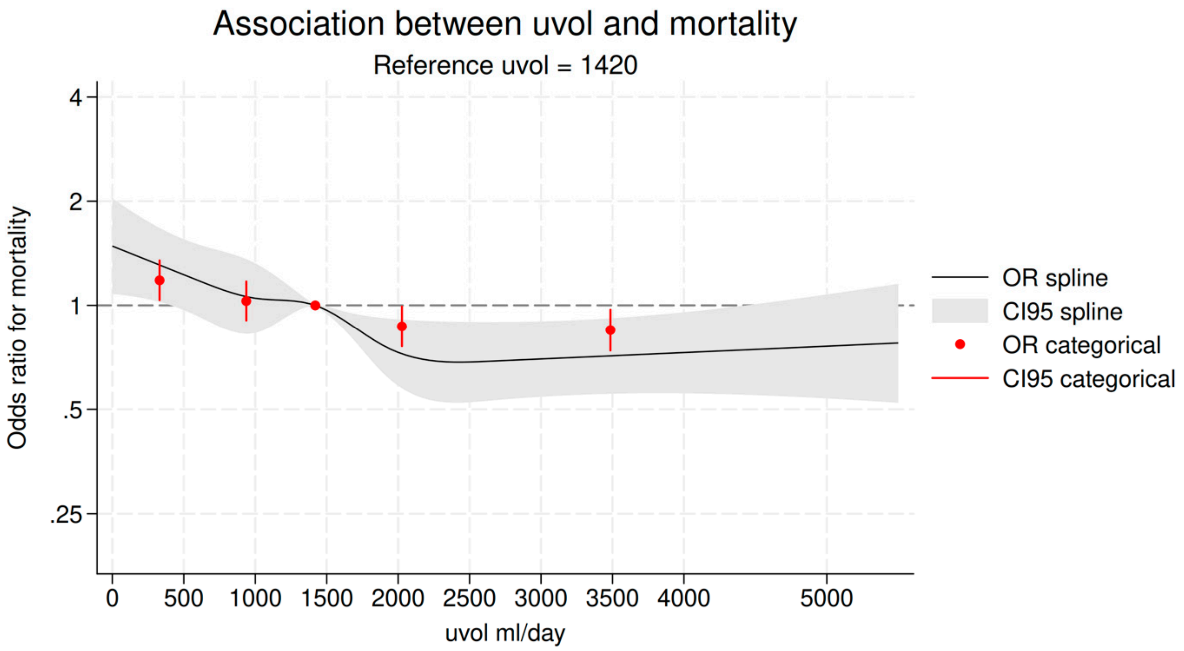

Figure S6. Association between urine volume and sixty-day hospital mortality when using restricted cubic splines for creatinine, urine volume and adjusted SAPS 2 score (n=14802)

This sensitivity multivariable analysis illustrates the association between urine volume and mortality while accounting for all other covariables (Region, Period, ICU Days, Sex, Age, BMI, Creatinine, adjusted SAPS 2, Reasons for ICU dependency) with the individual ICU as random effect. Reference urine volume, expressed as the mean when all covariables are in their reference groups, was 1420 ml/day in the multivariable model (n=14802). OR from treating urine volume continuously using restricted cubic splines (black line) is compared with OR from categorising urine volume (red dots).

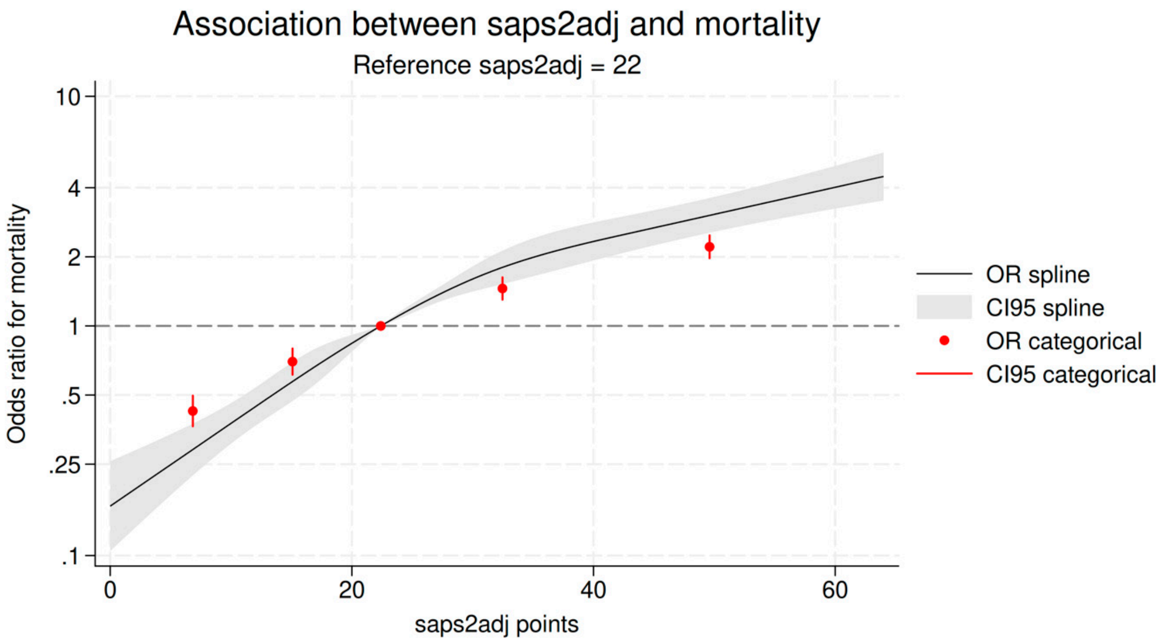

Figure S7. Association between adjusted SAPS 2 score and sixty-day hospital mortality when using restricted cubic splines for creatinine, urine volume and adjusted SAPS 2 score (n=14802)

This sensitivity multivariable analysis illustrates the association between adjusted SAPS 2 score and mortality while accounting for all other covariables (Region, Period, ICU Days, Sex, Age, BMI, Creatinine, Urine Volume, Reasons for ICU dependency) with the individual ICU as random effect. Reference adjusted SAPS 2, expressed as the mean [95% CI] when all covariables are in their reference groups, was 22 points in the multivariable model (n=14802). OR from treating adjusted SAPS 2 continuously using restricted cubic splines (black line) is compared with OR from categorising adjusted SAPS 2 (red dots).

		OR [95% CI] for RRT
Region	Europe	1.00 [1.00 1.00]
	North America	0.87 [0.63 1.19]
	Latin America	1.02 [0.82 1.25]
	Asia	1.11 [0.92 1.35]
Year	[2007-2011]	1.00 [1.00 1.00]
	[2012-2016]	1.11 [0.94 1.32]
	[2017-2021]	1.05 [0.86 1.28]
	[2022-2024]	1.01 [0.82 1.24]
ICU Days (days)	(0-1)	0.43*** [0.35 0.52]
	(2-3)	0.71*** [0.59 0.86]
	(4-6)	1.00 [1.00 1.00]
	(7-14)	1.20* [1.01 1.44]
	(15-..)	1.52*** [1.28 1.80]
	missing	0.80 [0.63 1.00]
Sex	female	1.00 [1.00 1.00]
	male	1.14* [1.02 1.27]
Age (years)	[18-30)	0.84 [0.64 1.12]
	[30-40)	0.99 [0.78 1.25]
	[40-50)	0.94 [0.77 1.14]
	[50-60)	1.06 [0.91 1.24]
	[60-70)	1.00 [1.00 1.00]
	[70-80)	0.76*** [0.66 0.87]
	[80-90)	0.54*** [0.44 0.65]
	[90...)	0.32*** [0.19 0.53]
BMI (kg/m^2)	[10-18.5)	0.98 [0.76 1.27]
	[18.5-25)	1.00 [1.00 1.00]
	[25-30)	1.17* [1.03 1.32]
	[30-35)	1.25** [1.06 1.47]
	[35-40)	1.30* [1.03 1.64]
	[40...)	1.07 [0.82 1.40]
	missing	0.89 [0.67 1.18]
Creatinine ICU Day 0	Q1:[0.1 - 0.7)	0.77* [0.61 0.99]
(mg/dl)	Q2:[0.7 - 0.9)	0.72** [0.56 0.92]
	Q3:[0.9 - 1.2]	1.00 [1.00 1.00]
	Q4:[1.2 - 1.8]	1.61*** [1.32 1.96]
	Q5:[1.8 - 15]	6.04*** [5.05 7.22]
	missing	2.70*** [2.17 3.36]
Urine Volume ICU Day 0	[0-670)	4.89*** [4.09 5.84]
(ml)	[670-1200)	1.51*** [1.24 1.84]
	[1200-1700)	1.00 [1.00 1.00]
	[1700-2460)	0.81 [0.65 1.01]
	[2460-9800)	0.73** [0.58 0.92]
	missing	4.24*** [3.44 5.22]
SAPS 2 adjusted	Q1	0.55*** [0.45 0.66]
	Q2	0.74*** [0.62 0.88]
	Q3	1.00 [1.00 1.00]

	Q4	1.21* [1.05 1.41]
	Q5	1.19* [1.02 1.39]
ICU dependency	no	1.00 [1.00 1.00]
	abdominal	1.25** [1.07 1.45]
	no	1.00 [1.00 1.00]
	burns	0.91 [0.45 1.85]
	no	1.00 [1.00 1.00]
	cardiac	1.68*** [1.46 1.93]
	no	1.00 [1.00 1.00]
	neurologic	0.55*** [0.46 0.66]
	no	1.00 [1.00 1.00]
	pulmonary	1.03 [0.91 1.17]
	no	1.00 [1.00 1.00]
	septic	1.75*** [1.54 1.99]
	no	1.00 [1.00 1.00]
	trauma	0.52*** [0.39 0.70]
	no	1.00 [1.00 1.00]
	other	1.72*** [1.48 2.00]
Constant		0.02*** [0.01 0.03]
Random intercept variance		1.73*** [1.52 1.97]
Observations		23485

Table S4. Associations between renal replacement therapy (RRT) and ICU- and patient-level factors in a multivariable logistic regression.

* p < 0.05, ** p < 0.01, *** p < 0.001

Odds ratios (OR) relative to the reference category (OR=1) are presented with 95% confidence intervals.

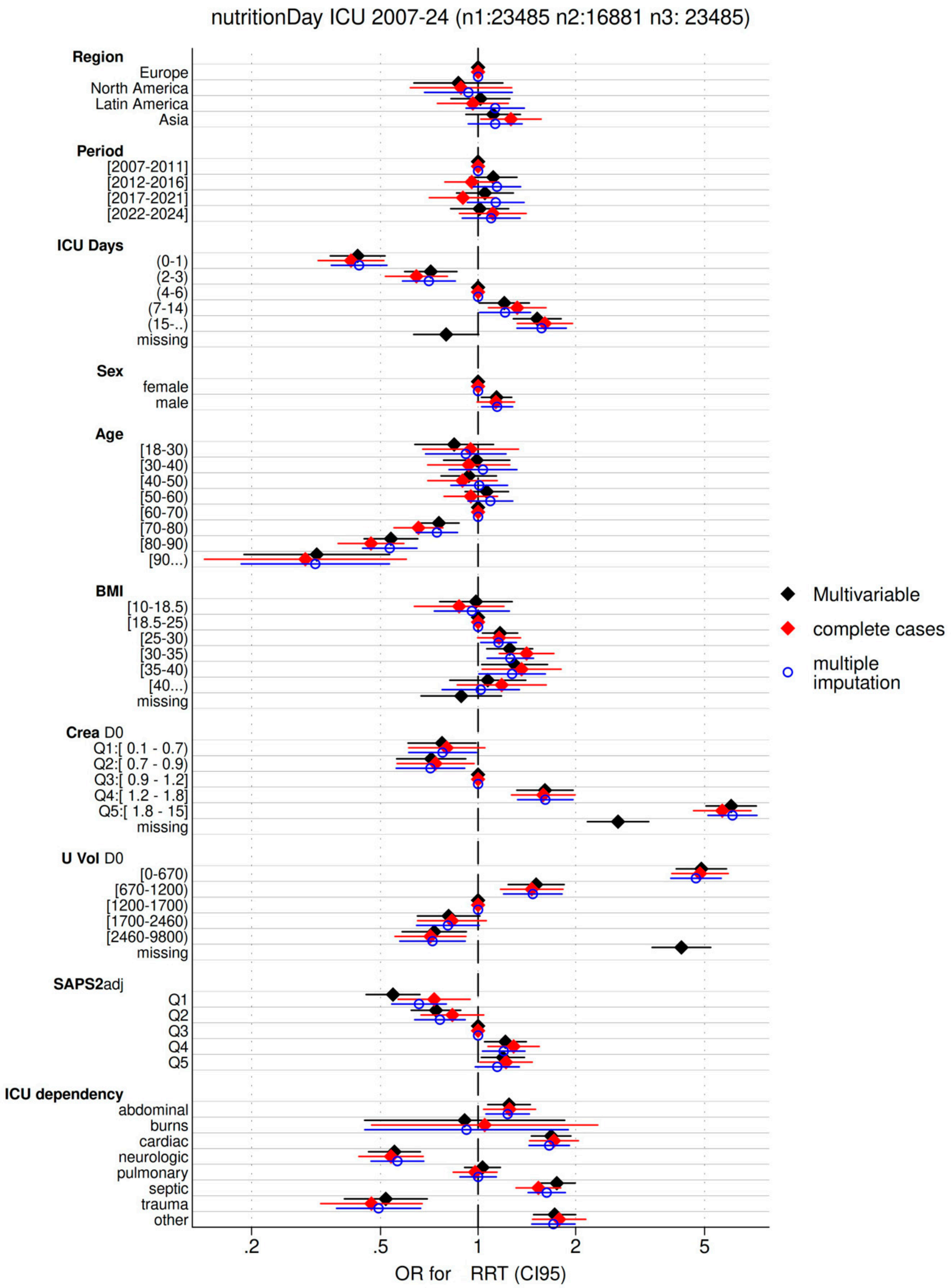

Figure S8. Sensitivity analysis for renal replacement therapy with complete cases excluding patients

with missing values for independent covariables (n2=16881 in red) or with imputation of missing values for independent covariables (n3=23485 in blue).

Missing values for independent covariables included missing ICU days, BMI, creatinine at ICU admission, urine volume at ICU admission, or more than three missing SAPS 2 components.

ICU Days in days; Age in years; BMI in kg/m^2; RRT: Renal Replacement Therapy; Crea D0: Creatinine at ICU admission in mg/dl; U Vol D0: Urine volume at ICU admission in ml; SAPS 2adj: Adjusted SAPS 2 score without age and urine-volume components

		Mean [95% CI] deviations of creatinine from baseline creatinine in mg/dl
Region	Europe	0.00 [0.00 0.00]
	North America	0.23*** [0.13 0.34]
	Latin America	-0.02 [-0.09 0.05]
	Asia	0.08* [0.02 0.14]
Year	[2007-2011]	0.00 [0.00 0.00]
	[2012-2016]	-0.07* [-0.13 -0.02]
	[2017-2021]	-0.18*** [-0.25 -0.11]
	[2022-2024]	-0.04 [-0.11 0.03]
Sex	female	0.00 [0.00 0.00]
	male	0.21*** [0.17 0.24]
Age (years)	[18-30)	-0.21*** [-0.29 -0.12]
	[30-40)	-0.18*** [-0.26 -0.10]
	[40-50)	-0.08* [-0.15 -0.01]
	[50-60)	-0.02 [-0.08 0.03]
	[60-70)	0.00 [0.00 0.00]
	[70-80)	0.04 [-0.01 0.09]
	[80-90)	0.09** [0.02 0.15]
	[90...)	0.00 [-0.15 0.15]
BMI (kg/m^2)	[10-18.5)	-0.10* [-0.19 -0.01]
	[18.5-25)	0.00 [0.00 0.00]
	[25-30)	0.05* [0.01 0.10]
	[30-35)	0.14*** [0.08 0.20]
	[35-40)	0.11** [0.03 0.20]
	[40...)	0.22*** [0.12 0.32]
	missing	0.01 [-0.09 0.10]
Urine Volume ICU Day 0	[0-670)	0.63*** [0.57 0.69]
(ml)	[670-1200)	0.10*** [0.04 0.16]
	[1200-1700)	0.00 [0.00 0.00]
	[1700-2460)	-0.04 [-0.09 0.02]

	[2460-9800)	-0.05 [-0.11 0.01]
	missing	0.48*** [0.41 0.56]
SAPS 2 adjusted	Q1	-0.19*** [-0.25 -0.13]
	Q2	-0.08** [-0.13 -0.02]
	Q3	0.00 [0.00 0.00]
	Q4	0.11*** [0.06 0.16]
	Q5	0.15*** [0.09 0.20]
ICU dependency	no	0.00 [0.00 0.00]
	abdominal	0.02 [-0.04 0.07]
	no	0.00 [0.00 0.00]
	burns	-0.19 [-0.40 0.03]
	no	0.00 [0.00 0.00]
	cardiac	0.11*** [0.06 0.16]
	no	0.00 [0.00 0.00]
	neurologic	-0.12*** [-0.18 -0.07]
	no	0.00 [0.00 0.00]
	pulmonary	-0.04 [-0.09 0.00]
	no	0.00 [0.00 0.00]
	septic	0.44*** [0.39 0.50]
	no	0.00 [0.00 0.00]
	trauma	-0.12** [-0.20 -0.05]
	no	0.00 [0.00 0.00]
	other	0.34*** [0.29 0.40]
Constant		1.07*** [0.98 1.17]
Random intercept variance		0.06*** [0.05 0.07]
Residual variance		1.66*** [1.62 1.69]
Observations		21054

Table S5. Mean deviations of serum creatinine at ICU admission from baseline creatinine for ICU- and patient-level factors in a multivariable linear regression.

* $p < 0.05$, ** $p < 0.01$, *** $p < 0.001$

Mean deviations are presented with 95% confidence intervals.

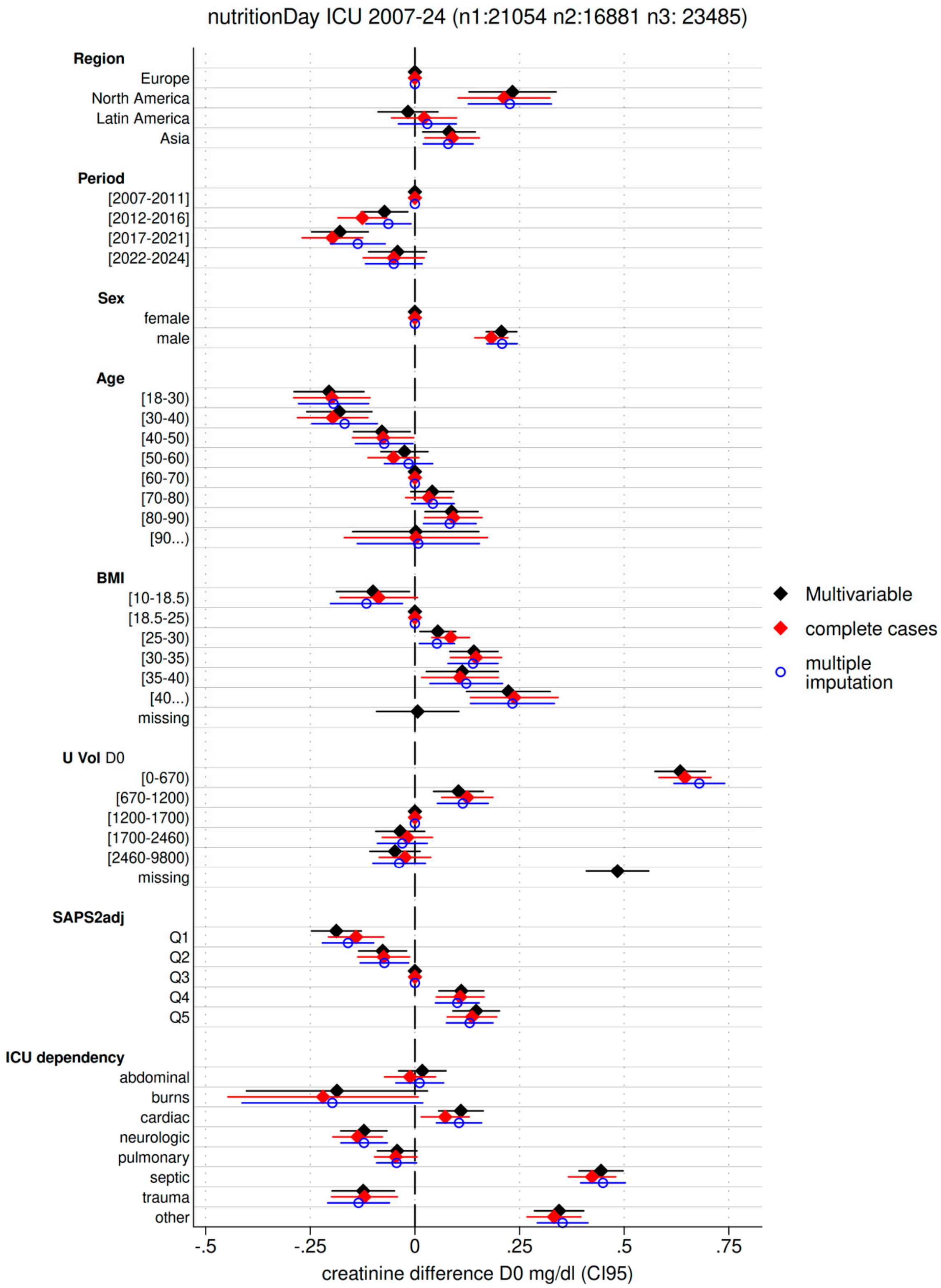

Figure S9. Sensitivity analysis for mean deviations of serum creatinine at ICU admission (D0) from baseline creatinine with complete cases excluding patients with missing values for independent

covariables (n2=16881 in red) or with imputation of both missing values for independent covariables and creatinine (n3=23485 in blue).

Mean deviations are presented with 95% confidence intervals. Baseline creatinine, expressed as the mean [95% CI] when all covariables are in their reference groups, was 1.07 [0.98-1.17] in the standard multivariable model (n1=21054 in black). Missing values for independent covariables included missing BMI, creatinine at ICU admission, urine volume at ICU admission, or more than three missing SAPS 2 components. Age in years; BMI in kg/m^2; U Vol D0: Urine volume at ICU admission in ml; SAPS 2adj: Adjusted SAPS 2 score without age and urine-volume components

		Mean [95% CI] deviations of urine volume from baseline urine volume in ml
Region	Europe	0.00 [0.00 0.00]
	North America	-360.28*** [-491.97 -228.59]
	Latin America	-345.66*** [-433.85 -257.47]
	Asia	-90.51* [-167.70 -13.32]
Year	[2007-2011]	0.00 [0.00 0.00]
	[2012-2016]	-97.63*** [-155.24 -40.02]
	[2017-2021]	-267.90*** [-338.38 -197.41]
	[2022-2024]	-223.79*** [-299.17 -148.41]
Sex	female	0.00 [0.00 0.00]
	male	108.08*** [76.95 139.21]
Age (years)	[18-30)	317.59*** [247.52 387.66]
	[30-40)	215.56*** [150.13 281.00]
	[40-50)	172.53*** [115.81 229.24]
	[50-60)	65.62** [18.48 112.76]
	[60-70)	0.00 [0.00 0.00]
	[70-80)	-60.94** [-103.76 -18.12]
	[80-90)	-156.06*** [-210.20 -101.93]
	[90...)	-249.01*** [-378.56 -119.46]
BMI (kg/m^2)	[10-18.5)	-217.80*** [-291.17 -144.44]
	[18.5-25)	0.00 [0.00 0.00]
	[25-30)	23.24 [-12.56 59.05]
	[30-35)	29.23 [-19.47 77.93]
	[35-40)	4.44 [-68.15 77.03]
	[40...)	175.18*** [91.43 258.94]
	missing	-78.38 [-169.94 13.18]
Creatinine ICU Day 0	Q1:[0.1 - 0.7)	44.59 [-5.74 94.92]
(mg/dl)	Q2:[0.7 - 0.9)	2.90 [-46.07 51.86]

	Q3:[0.9 - 1.2]	0.00 [0.00 0.00]
	Q4:[1.2 - 1.8]	-90.82*** [-139.21 -42.43]
	Q5:[1.8 - 15]	-349.28*** [-399.20 -299.35]
	missing	-205.53*** [-272.30 -138.76]
SAPS 2 adjusted	Q1	-60.83* [-112.34 -9.33]
	Q2	-32.54 [-80.56 15.48]
	Q3	0.00 [0.00 0.00]
	Q4	-21.32 [-66.28 23.64]
	Q5	-4.38 [-51.29 42.53]
ICU dependency	no	0.00 [0.00 0.00]
	abdominal	-85.34*** [-134.43 -36.26]
	no	0.00 [0.00 0.00]
	burns	57.06 [-131.30 245.41]
	no	0.00 [0.00 0.00]
	cardiac	47.18* [0.88 93.49]
	no	0.00 [0.00 0.00]
	neurologic	157.33*** [109.77 204.88]
	no	0.00 [0.00 0.00]
	pulmonary	0.27 [-40.29 40.83]
	no	0.00 [0.00 0.00]
	septic	-62.17** [-107.50 -16.85]
	no	0.00 [0.00 0.00]
	trauma	173.21*** [109.31 237.12]
	no	0.00 [0.00 0.00]
	other	-87.51*** [-138.09 -36.94]
Constant		1872.03*** [1784.29 1959.76]
Random intercept variance		179466.40*** [156369.32 202563.48]
Residual variance		1131420.45*** [1109042.76 1153798.14]
Observations		20753

Table S6. Mean deviations of urine volume at ICU admission from baseline urine volume for ICU- and patient-level factors in a multivariable linear regression.

* p < 0.05, ** p < 0.01, *** p < 0.001

Mean deviations are presented with 95% confidence intervals.

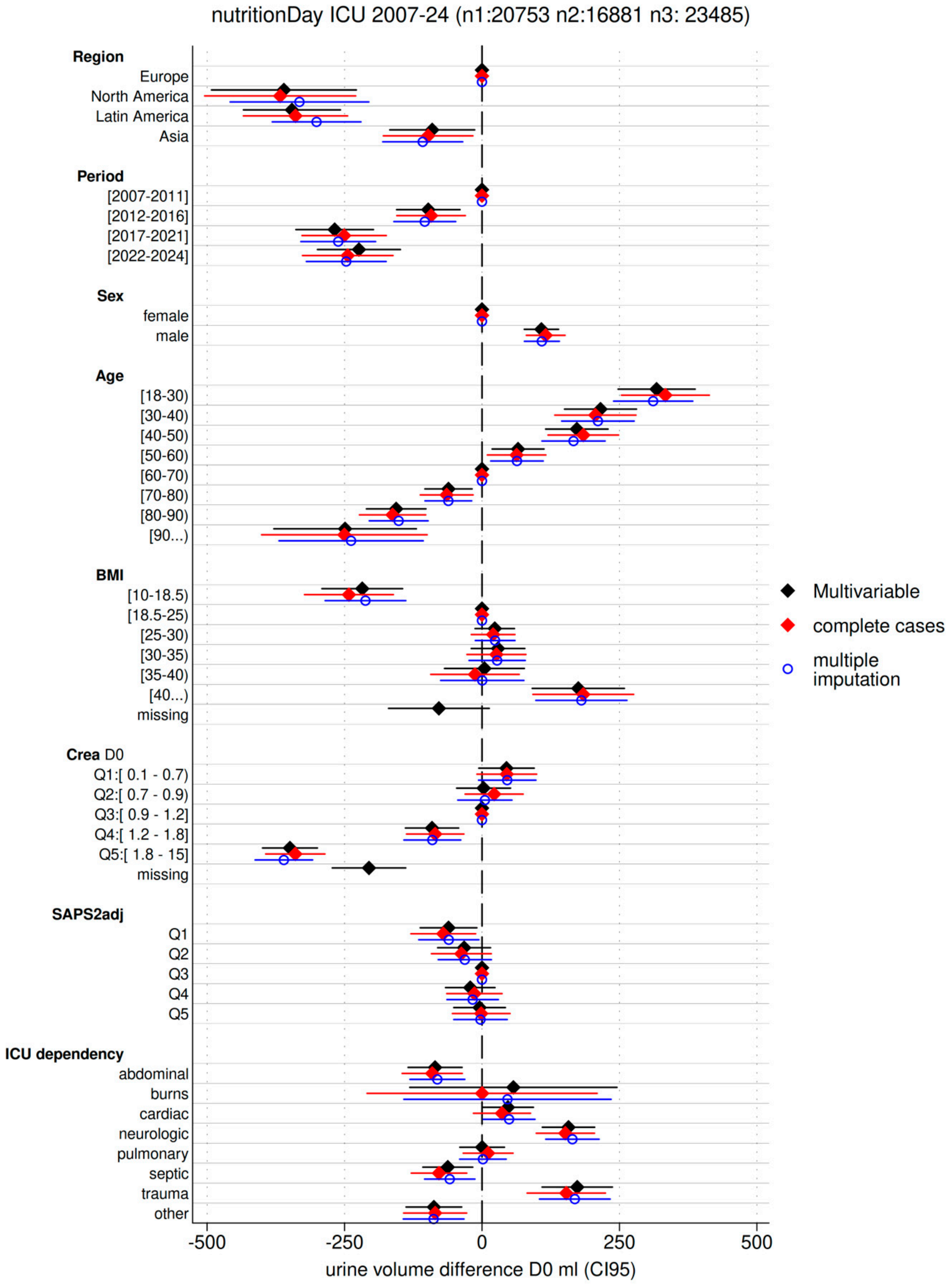

Figure S10. Sensitivity analysis for mean deviations of urine volume at ICU admission (D0) from baseline urine volume with complete cases excluding patients with missing values for independent

covariables (n2=16881 in red) or with imputation of both missing values for independent covariables and urine volume (n3=23485 in blue).

Mean deviations are presented with 95% confidence intervals. Baseline urine volume, expressed as the mean [95% CI] when all covariables are in their reference groups, was 1872 [1784-1960] ml.

Missing values for independent covariables included missing BMI, creatinine at ICU admission, urine volume at ICU admission, or more than three missing SAPS 2 components.

Age in years; BMI in kg/m^2; SAPS 2adj: Adjusted SAPS 2 score without age and urine-volume components

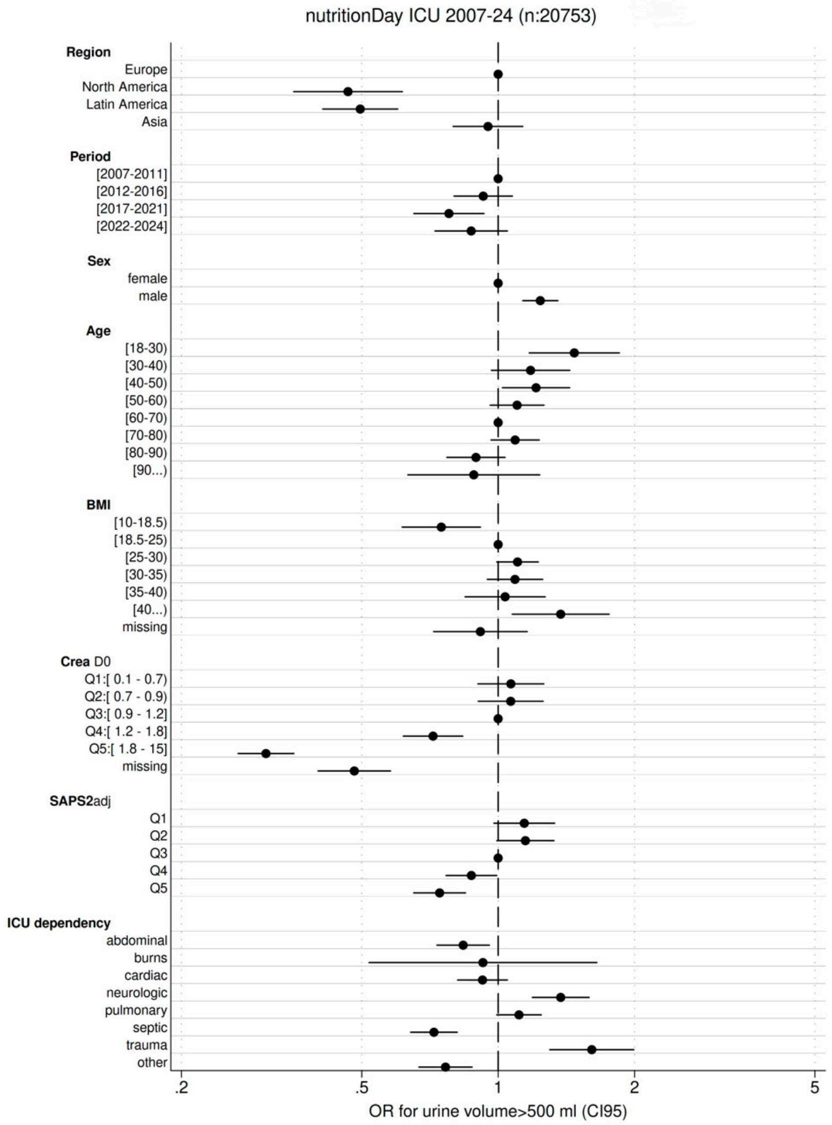

Figure S11. Sensitivity analysis for higher versus lower urine volume (≥500 vs <500 ml) at ICU admission in a multivariable mixed-effects logistic regression

Urine volume was categorised into < and ≥ 500 ml.

OR: Odds ratio; Age in years; BMI in kg/m^2; Crea D0: Creatinine at ICU admission in mg/dl; SAPS 2adj: Adjusted SAPS 2 score without age and urine-volume components.
